# Effect of Natural and Semisynthetic Pseudoguianolides on the Stability of NF-κB:DNA Complex Studied by Agarose Gel Electrophoresis

**DOI:** 10.1371/journal.pone.0115819

**Published:** 2015-01-23

**Authors:** Rodrigo Villagomez, Rajni Hatti-Kaul, Olov Sterner, Giovanna Almanza, Javier A. Linares-Pastén

**Affiliations:** 1 Centre for Analysis and Synthesis, Lund University, P.O. Box 124, 221 00 Lund, Sweden; 2 Instituto de Investigaciones Químicas, Facultad de Ciencias Puras y Naturales, Universidad Mayor de San Andrés, P.O. Box 303 La Paz, Bolivia; 3 Biotechnology, Dept. of Chemistry, Lund University, P.O. Box 124, SE-22 100 Lund, Sweden; University of Quebect at Trois-Rivieres, CANADA

## Abstract

The nuclear factor κB (NF-κB) is a promising target for drug discovery. NF-κB is a heterodimeric complex of RelA and p50 subunits that interact with the DNA, regulating the expression of several genes; its dysregulation can trigger diverse diseases including inflammation, immunodeficiency, and cancer. There is some experimental evidence, based on whole cells studies, that natural sesquiterpene lactones (Sls) can inhibit the interaction of NF-κB with DNA, by alkylating the RelA subunit via a Michael addition. In the present work, 28 natural and semisynthetic pseudoguianolides were screened as potential inhibitors of NF-κB in a biochemical assay that was designed using pure NF-κB heterodimer, pseudoguianolides and a ~1000 bp palindromic DNA fragment harboring two NF-κB recognition sequences. By comparing the relative amount of free DNA fragment to the NF-κB – DNA complex, in a routine agarose gel electrophoresis, the destabilizing effect of a compound on the complex is estimated. The results of the assay and the following structure-activity relationship study, allowed the identification of several relevant structural features in the pseudoguaianolide skeleton, which are necessary to enhance the dissociating capacity of NF-κB–DNA complex. The most active compounds are substituted at C-3 (α-carbonyl), in addition to having the α-methylene-γ-lactone moiety which is essential for the alkylation of RelA.

## Introduction

The nuclear factor κB (NF-κB) is considered to be a promising target for drug discovery. It regulates the transcription of pro-inflammatory and anti-apoptotic proteins, among others; dysregulation can lead to the development of chronic inflammation, immunodeficiency and cancer. Hence, NF-κB plays an important role in oncogenesis, proliferation and cancer metastasis [1–6]. The NF-κB regulation pathway is complex but has been thoroughly investigated, and involves several steps [7,8] in addition to advanced “crosstalk” with other signaling pathways [9]. In practice, two major pharmaceutical approaches have received most attention: a) inhibition of the proteolytic activity of proteasome 26S, and b) inhibition of κB protein kinase (IKK). Both enzyme activities are indispensable for the degradation and phosphorylation of inhibitor-κB protein (IκB), and consequently for the activation of NF-κB [10–12].

Several steps in the NF-κB activation pathway can be evaluated via biochemical and cell-based assays. The transcription capacity of the factor can be monitored by different methods and those mostly used are based on reporter gene assays. Examples are the firefly luciferase gene (LUC) [13,14], secreted embryonic alkaline phosphatase (SEAP) [15], chloramphenicol acetyltranferase (CAT) [16] and β-galactosidase [17]. The binding of NF-κB to DNA can be studied with Electrophoretic Mobility Shift Assays (EMSA) by polyacrylamide gel electrophoresis (PAGE) [18], and recently a highly sensitive biochemical method based on luminescent switch-on probe was reported [19]. Other methods are more specific for the early stages of the pathway, e.g. the degradation of IκB using a inhibitory protein labeled with a fragment of β-galactosidase (ProLabel) [20], and monitoring the nuclear translocation of the activated NF-κB by fluorescence cytometry [21]. Western blot is usually used to determine IKK activation as well as the degree of phosphorylation and degradation of the IκB protein [22–24]. Nevertheless, majority of these techniques are either expensive or time demanding, and most of them are performed in whole cells. Biochemical assays allow a direct study of interactions between NF-κB and potential inhibitors providing insights of structure—activity relationships (SAR), which is the basis for the rational design of new drugs. In addition, the low cost and fast assays allow screening of large number of inhibitor candidates.

**Figure 1:**
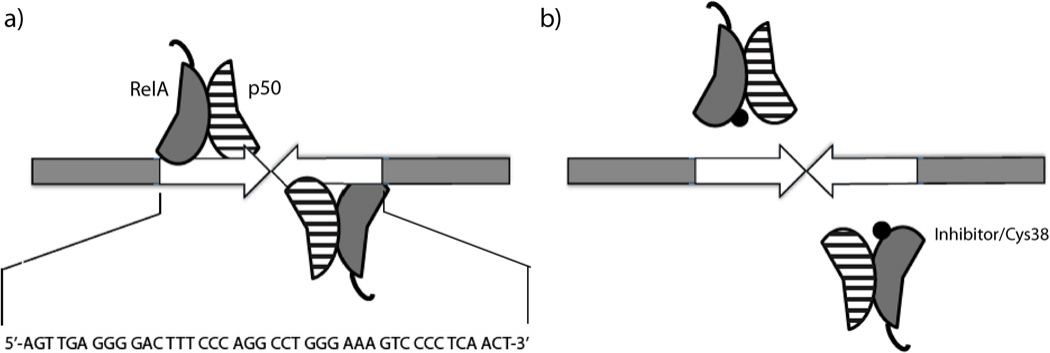
Schematic view of interactions. **a)** Complexation between the palindromic DNA-recognition target and two RelA/p50 heterodimers. **b)** Effect of inhibitors on RelA (cys38) hindering the formation of the complex.

**Figure 2:**
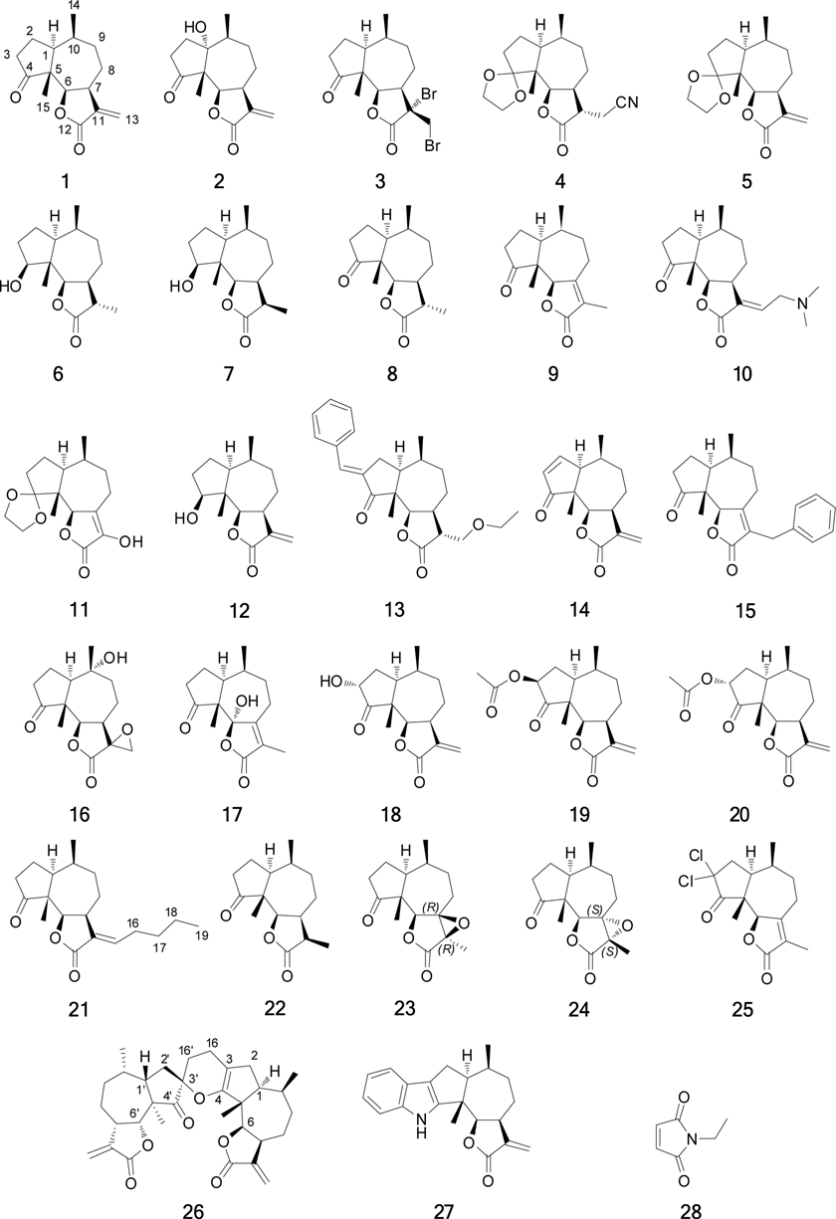
Compounds tested as inhibitors of the NF-κB:DNA-recognition target complex formation.

Several natural products are potential lead structures for the development of NF-κB inhibitors, and an important group is the sesquiterpene lactones (SLs) [25,26]. Almost all the experimental evidence demonstrates that the SLs act as alkylating agents by a Michael addition mechanism, and an especially interesting target is residue cys-38 in the NF-κB transcriptional subunit RelA (canonical pathway), as the formation of the complex of NF-κB with DNA is prevented if the thiol group of this cysteine is alkylated [27,28]. Michael acceptors as well as other alkylating agents have traditionally been associated with toxicity, but the possibility to increase their selectivity makes them interesting alternatives for drug discovery [29]. Based on this knowledge, we developed a biochemical assay to study the effects of SLs on the interaction between a designed DNA-recognition target (fragment ~1000 bp containing two κB-recognition sites oriented in a palindrome way), and the canonical NF-κB heterodimer (RelA/p50) (Fig. 1). Both interactions, NF-kB:DNA and NF-kB:DNA:SLs, were observed by agarose gel electrophoresis. The evaluated SLs were derivatives of damsin (**1**) (Fig. 2), a natural product with a recognized NF-κB inhibitory capacity [30]. In total 27 compounds were tested, representing a substantial structural diversity, facilitating an initial SAR analysis.

## Materials and Methods

### Chemicals

All chemicals of analytical grade were purchased from different commercial suppliers and were used without further purification unless otherwise stated.

### Strains and Plasmids

*Escherichia coli* NovaBlue and *E. coli* BL21(DE3) strains for cloning and expression, respectively, were purchased from Novagen. Both strains were cultivated in Luria-Bertani (LB) broth and LB-16% agar. Cloning vector pUC19 was purchased from Thermo Scientific and the co-expression vector pCOLADuet was from Novagen. Ampicillin (100 µg/mL) was added to the medium to select the *E. coli* NovaBlue harboring pUC19 derivative constructs, and 30 µg/mL kanamicyn was included for the selection of *E. coli* BL21(DE3) harboring pCOLADuet derivative constructs.

### Cloning of RelA and p50 Genes

The strategy used was focused on the co-expression of the RelA/p50 heterodimer, with a 6X histidine tag in the N-terminal end of the RelA subunit for an effective purification of soluble heterodimer, similar to the one reported previously [40,41]. Synthetic genes to overexpress the truncated human RelA (residues 20–291) and p50 (residues 42–353) in *E. coli* were designed. The codon optimization was performed using the Optimizer Server (*http://genomes.urv.es/OPTIMIZER/*) and the analysis of the optimal codons by *E. coli* Codon Usage Analyzer 2.1 (*www.faculty.ucr.edu/~mmaduro/codonusage/usage.htm*). Every optimized gene sequence was synthesized and inserted into pUC57 vectors (GeneScript USA Inc). The genes were amplified individually from pUC57 using the following primers: RelA-Forward (5’-GGA TCC GGC CTA CGT TGA AAT CAT CGA ACA GCC GAA ACA GC-3’), RelA-Reverse (5’- GCG GCC GCG TCC GGC AGG TAC TGG AAT TCC ATC GG-3’), p50-Forward (5’- CAT ATG GGT CCG TAC CTG CAG ATC CTG GAA CA-3’) and p50-Reverse (5’- TTA ATT AAG CTT CCG GGT AGT ACA GGA ACG GTT TCG G-3’). The PCR products were inserted individually into pUC19/*sma*I vector for proliferation and finally subcloned into the *Bam*HI/*Not*I (RelA) and *Pac*I/*Nde*I (p50) sites of the co-expression vector pCOLADuet.

### Expression and Purification of the Proteins

RelA/p50 heterodimer was co-expressed in *E. coli* BL21(DE3). The cells were grown in shake flasks at 30°C in LB medium supplemented with 25 µg/ml kanamycin. The induction was done with 1 mM isopropyl β-D-thiogalactopyranoside (IPTG) when the optical density reached 0.6 at 600 nm, followed by overnight incubation at room temperature with shaking at 100 rpm. The cells were harvested by centrifugation (9300 g for 20 min), re-suspended in binding buffer (20 mM HEPES and 500 mM NaCl at pH 7.4) and disrupted by sonication (10 x 2 min pulses with 2 min interval). The cell lysate was centrifuged for 20 minutes at 22000 rpm and the supernatant was used for protein purification. The recombinant proteins were purified by affinity chromatography using 1 mL Histrap HP column with 20 mM HEPES, 500 mM NaCl pH 7.4 as the binding buffer, and the buffer with 500 mM imidazole as the elution buffer. The column fractions were analyzed by SDS-PAGE, and imidazole was removed by dialysis against 2 x 1 L of binding buffer.

### Construction of the DNA Recognition Target

A DNA fragment of ~1000 bp containing NF-κB recognition sequences was constructed as follows: a palindromic synthetic oligonucleotide (NF-κB target) AGT TGA GGG GAC TTT CCC AGG CCT GGG AAA GTC CCC TCA ACT was inserted in pUC19/SmaI vector. Subsequently, a fragment containing the palindromic sequence (NF-κB target) in the middle was generated by PCR using DreamTaq PCR Master Mix (Thermo Scientific). The primers used were κB-Forward GAA GGC AAA ATG CCG CAA AAA AGG and κB-Reverse GCG TCG ATT TTT GTG ATG CTC GTC. The mixture was incubated for 2 min at 98°C, 35 amplification cycles were performed (denaturation for 20 s at 98°C, annealing for 30 s at 60°C, and extension for 40 s at 72°C) and this was followed by a final extension for 7 min at 72°C. The quality of the product was confirmed by electrophoresis on 1% (w/v) agarose gel and visualization with GelRed Nucleic Acid Gel Stain (Invitrogen, USA).

### Determination of NF-κB:DNA Recognition Target Interaction and Optimal Molar Ratio

The reaction was carried out in a volume of 10 μL for 2 h at 37°C. The protein:DNA molar ratio was varied in three different ranges: **1. From 2:1 to 13:1:** using 1.5 μL of DNA target (85 ng/μL) the amount of NF-κB (28 ng/μL) was varied from 1 μL (ratio 2:1) to 6 μL (13:1), **2. From 19:1 to 112:1:** to 1.0 μL of DNA target (85 ng/μL) was added varying volumes of NF-κB dilution (165 ng/μL) from 1 μL (19:1) to 6 μL (112:1), **3. From 298:1 to 669:1:** with 1.0 μL of DNA target (85 ng/μL) the amount of NF-κB (660 ng/μL) was varied from 4 μL (298:1) to 9 μL (669:1). The volumes of all the reaction mixtures were made up to 10 μL with nuclease-free water. Two blanks were prepared, the first one with HEPES (B1) with 1 μL of DNA target (100 ng/μL) in 9 μL of HEPES buffer (20 mM HEPES and 500 mM NaCl at pH 7.4), and the second one without HEPES (B2): 1 μL of DNA target (100 ng/μL) in 9 μL of nuclease-free water. The range from 298:1 to 669:1 was performed in triple replicates including the blanks. The samples were analyzed by electrophoresis on 1% agarose gel (TAE 1X; 100V for 1.5 h) and visualized with GelRed Nucleic Acid Gel Stain. Quantification of the relative DNA concentration on the agarose gels was performed using Bio-Imaging Systems Mini Bis Pro (Dual UV configuration 254–365 nm), software GelQuant Pro v11.4.

### Determination of the Optimal Inhibitor Concentration and Reaction Time

A factorial experiment was performed in a reaction volume of 10 μL, keeping the DNA:protein ratio constant, and varying the inhibitor concentration and reaction time. For this, four stock solutions of **1** in DMSO were prepared: 30 mM, 60 mM, 90 mM and 120 mM. Then 1 μL of each solution were added to four aliquots of pure NF-κB (8 μL, 660 ng/μL). The four reactions mixtures were left for 4 h at 37°C. Subsequently, 1 μL of DNA target (85 ng/μL) was added and the mixture was incubated at 37°C for 2 more hours. The procedure was repeated with reaction times of 3, 2 and 1 h, respectively, between the inhibitor and NF-κB. In total, 16 experiments were done.

### Determining the Effect of Different Inhibitors and Their Solubility in the System

All the experiments were performed in triplicates in reactions, with 8 μL of NF-kB and 1 μL of inhibitor in a total volume of 10 μL. The mixture was incubated for four hours at 37°C, after which 1 μL of DNA target dissolution was added and incubated for two more hours at 37°C. Four different diluted reaction mixtures were prepared from the following solutions: 85 ng/μL DNA target, 660 ng/μL NF-kB and 90 mM inhibitors (compounds 1 and 4 dissolved in DMSO), giving different DNA:NF-κB molar ratios (Table A in S2 File). Thus, both the inhibitor and NF-κB were diluted in factors of 1, 3/4, 1/2 and 1/4, while the DNA target in factors 1, 1/2, 1/4 and 1/8.

### Effect of DMSO

Reaction mixtures with 10%, 20% and 30% v/v DMSO were prepared. The addition of extra volume (2 and 3 μL) of DMSO to reach 20% and 30% forced to decrease the volume of NF-κB (7 and 6 μL) giving different final concentrations (528, 462 and 396 ng/μL respectively). Then, 1 μL of inhibitor was added in each solution to give a final concentration of 9 mM. These pre-mixtures were incubated for 4 hours at 37°C, then 1 μL of DNA target was added (giving a final concentration of 8.5 ng/μL) and the mixtures were incubated for two more hours at 37°C (Table B in S2 File).

### Synthesis of Pseudoguianolides

Following pseudoguianolides were synthesized according to the protocols described in S1 File: (6S,9aR,9bR)-3-hydroxy-6,9a-dimethyl-4,5,6,6a,7,8,9a,9b-octahydro-2H-spiro[azuleno[4,5-b]furan-9,2′-[1,3]dioxolan]-2-one **(11)**; 3β-acetoxydamsin **(19)**; (E)-13-n-Butildamsin **(21)**; (7*R*, 11*R*)-Epoxy-13-hydrodamsin and (7*S*, 11*S*)-Epoxy-13-hydrodamsin **(23 and 24)**; 3,3-Dichlorodamsin **(25)**; (3aS,3a′S,6S,6′S,8S,9aR,9bR,11b′R,11c′R)-6,6′,9a,11b′-tetramethyl-3,3′-dimethylene- 3a,3a′,4,4′,5,5′,6,6a,6′,6a′,7,7′,8′,9′-tetradecahydro-2H-spiro[azuleno[4,5-b]furan-8,10′-furo[3′,2′:7,8]azuleno[1,2-b]pyran]-2,2′,9(3H,3′H,9aH,9bH,11b′H,11c′H)-trione **(26)** and (E)-13-Bromodamsin **(29)**.

### Structure Determination

HMRS (ESI) spectra of the pseudoguianolides were recorded with a Micromass Q-TOF Micro spectrometer. NMR spectra (in CDCl_3_) were recorded using a Bruker DRX 400 MHz at 400 Mhz (^1^H) and at 100 MHz (^13^C) and Bruker DRX 500 MHz at 500 MHz (^1^H) and 125 MHz (^13^C). Chemical shifts are given in ppm relative to TMS using the residual CHCl_3_ in CDCl_3_ solution as internal standard (7.25 ppm ^1^H and 77.00 ppm ^13^C). All flash chromatography was performed with 60 Å 30–75 μm Silica gel. TLC analyses were made on Silica Gel 60 F254 (Merck) plates.

## Results and Discussion

### Determination of NF-κB /DNA Complex Formation and Optimal Molar Ratio

A well-known κB-recognition site (5’-TGGGGACTTTCC-3’) [31] was selected for the design of a palindromic sequence, which was inserted exactly in the middle of a ~1000bp DNA fragment, in order to have a clear DNA band in the routine agarose (1%) electrophoresis. This was done with the assumption that palindromic sequences could enhance the binding of two RelA/p50 heterodimers with one DNA molecule to obtain a higher molecular weight NF-κB-DNA complex (Fig. 1). The response is the quantified relative concentration (%) of the free DNA recognition target on the agarose gel.

**Figure 3:**
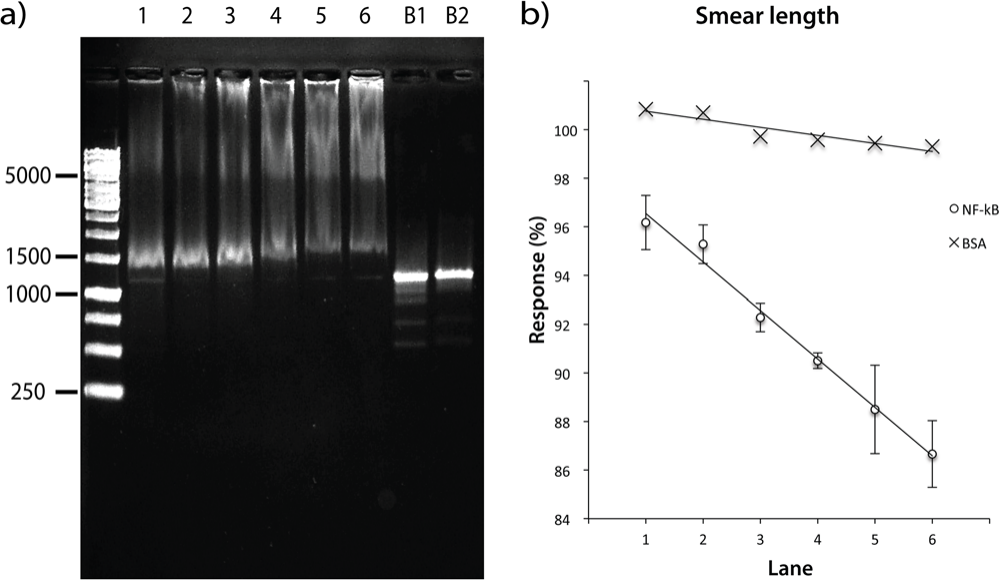
Optimization of Molar Ratio NF-κB-DNA. **a)** Agarose gel showing the effect of NF-κB on the palindromic DNA-recognition target. NF-κB-DNA molar ratio per Lane: 1 (253:1), 2 (316:1), 3 (379:1), 4 (443:1), 5 (506:1), 6 (569:1), B1 (Blank with HEPES Buffer) and B2 (Blank with nuclease-free water). **b)** (′) Standard addition curve for each molar ratio of NF-κB:DNA target (as lane number), y = -0.0199x + 0.9854, R^2^; = 0.98911; (**X**) Standard addition curve for each molar ratio of Bovine serum albumin:DNA target (as lane number).

In order to optimize the protein:DNA molar ratio, two responses were measured; the concentration of free DNA-recognition target (that was not complexed by NF-κB) and the total length of smear. The response was measured for molar ratios ranging from 2:1 to 669:1. The experiment was performed in triplicates (Fig. 3a). Ratios higher than 298:1 affected significantly the normal mobility of the DNA band (the concentration was reduced to a minimum of 4±2%). In order to demonstrate that NF-κB is the only component in the mixture that affects the DNA mobility, two blanks were included, the first with HEPES buffer (used in the protein solutions) and the second with nuclease-free water (used for DNA dilutions); neither interfered with the DNA mobility. On the other hand, the smear length of the DNA recognition target was demonstrated to be linearly dependent on the NF-κB protein concentration as shown in Fig. 3b. In addition, when the same experiment was repeated using bovine serum albumin protein (BSA), no effect was observed on the DNA mobility, ruling out the high concentration of an unrelated protein as being responsible for the DNA retention (Fig. 3b). The results of these experiments suggested that best condition to observe a strong interaction between NF-κB and the DNA recognition target is NF-κB:DNA molar ratio of 595:1 (protein concentration 528 ng/μL and DNA concentration 8.5 ng/μL).

### Optimization of Inhibitor Concentration and Reaction Time

**Figure 4:**
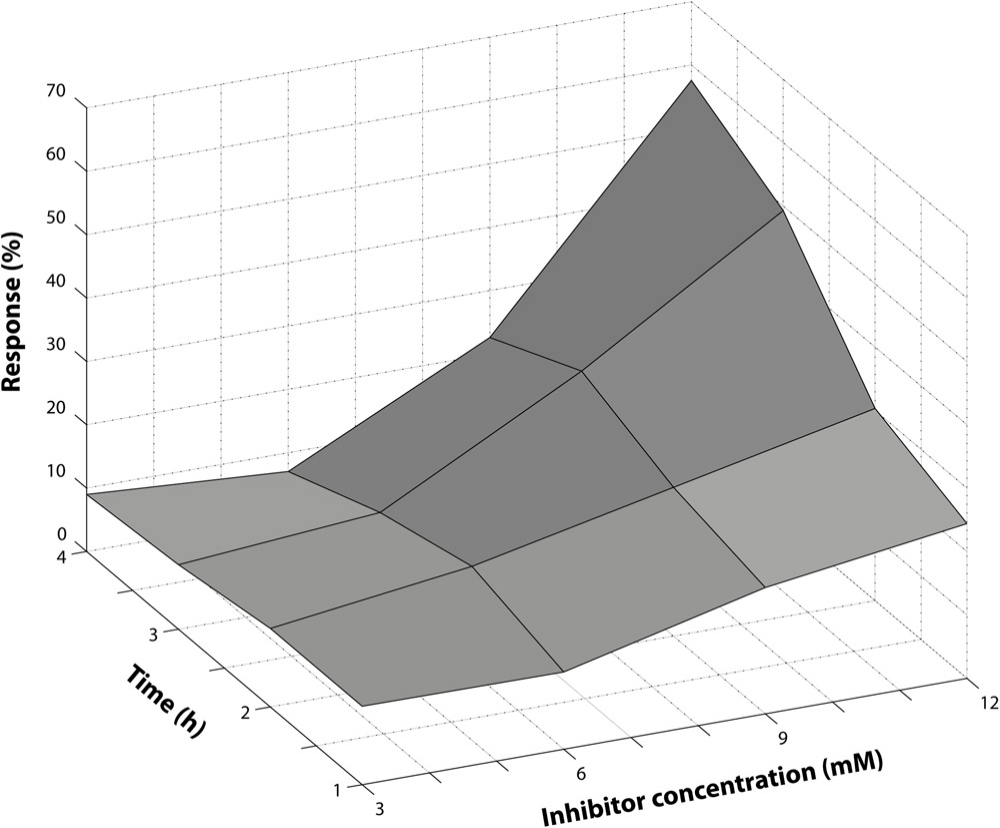
Optimization of inhibitor concentration and reaction time. Reaction of NF-κB with 3–12 mM inhibitor (**1**) at 1–4 h reaction time. The vertical axis represents the response as the percentage of released DNA target compared to the control (pure DNA target at 8.5 ng/μL). The coordinates show the concentration of damsin (**1**) (inhibitor) and the reaction time between NF-κB and the inhibitor.

Damsin **(1)** (Fig. 2) is a known inhibitor of NF-κB [30] and was used for the semisynthesis of several compounds tested in this study. In order to determine the optimal inhibitor concentration and reaction time, a combination of different damsin concentrations and different incubation times with NF-κB were evaluated. The response measured was the percentage of DNA released with respect to the control (8.5 ng/μL DNA in nuclease free water) (Fig. 4). The protein:DNA molar ratio was fixed at 595:1 (see previous section). At the lowest concentration of inhibitor (3 mM) the response did not change with time, giving a constant value of DNA released (about 10%). On the other hand, at the highest inhibitor concentration (12 mM), the response has shown a polynomial trend as a function of time, reaching a maximum of 60% after 4 h. However, a relatively lower response is preferred for the detection of highly active compounds or to neglect compounds with poor activity. Thus responses between 20 to 25% were considered as the most adequate for testing a range of damsin **(1)** derivatives, corresponding to 9 mM damsin concentration and 4 h incubation time at 37°C. An additional benefit of the selected inhibitor concentration is the constant response between 3 and 4 h of reaction. The same concentration was used for the positive control in further experiments.

### Determination of Activity and Solubility of the Compounds in the Reaction System

**Figure 5:**
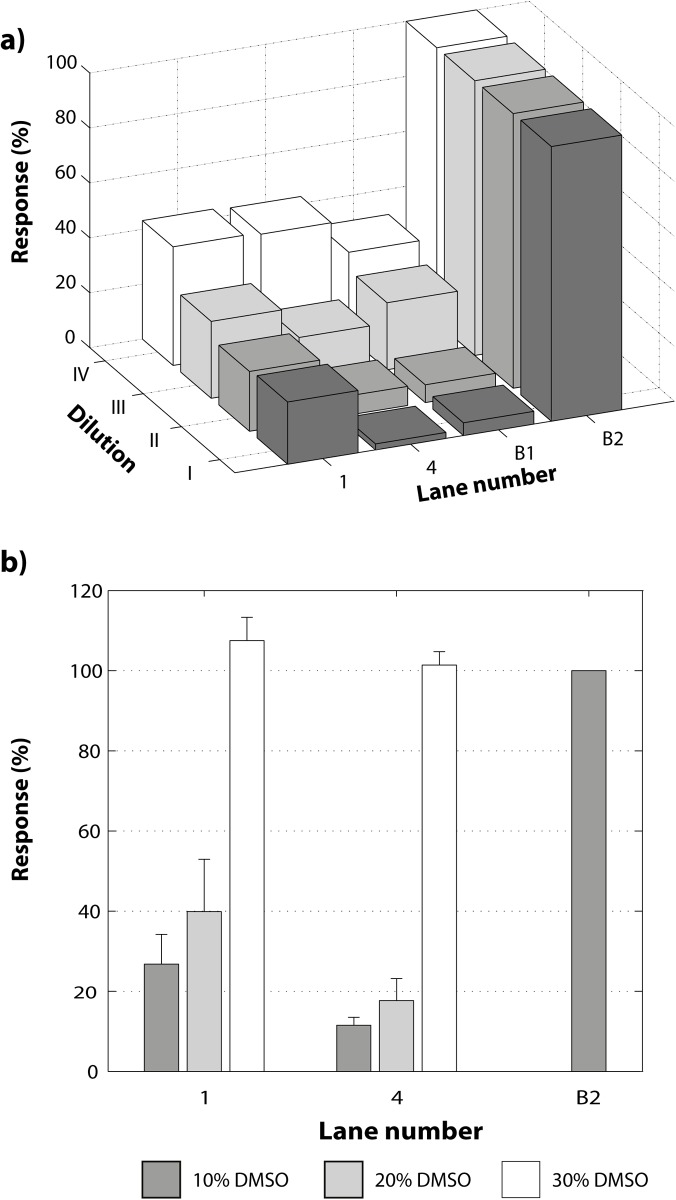
Effect of dilution and DMSO concentration on the interaction of NF-κB/DNA complex with the inhibitors. **a)** Effect of 1) active compound **1**; 2) inactive compound **4**; B1) control complex NF-κB:DNA target and B2) free DNA target, at four different dilutions of the system: I) Initial conditions; II) Inhibitor at ¾, NF-κB at ¾ and DNA target to ½ of initial concentration; III) Diluting inhibitor to ½, NF-κB to ½ and DNA target to ¼ of initial concentration and IV) Diluting inhibitor at ½, NF-κB at ½ and DNA target at ¼ of initial concentration. **b)** Effect of DMSO concentration on the response for two different inhibitors compared with one control: 1) compound **1**; 2) inactive compound **4**; B2) Free DNA target. The concentration of DMSO was increased at the same time as the protein concentration was decreased as follows: 10% DMSO (Protein:DNA molar ratio of 595:1); 20% DMSO (Protein:DNA molar ratio of 521:1) and 30% DMSO (Protein:DNA molar ratio of 446:1).

With the aim to evaluate the effect of active and non-active compounds on the NF-κB:DNA complex stability, two compounds were selected: **1**, that was used in the optimization process, which has a α-methylene-γ-lactone moiety responsible for the alkylating capacity and compound **4**, lacking the α-methylene-γ-lactone moiety and therefore presumed to be inactive. Furthermore, the sample solubility is a crucial factor in the development of biochemical and biological assays. Usually a co-solvent is necessary and the most frequently used is DMSO [32]. Hence, the effect of the selected compounds was tested at the same time as their solubility. In Fig. 5a, different responses for **1** and **4** were observed and, as expected, compound **1** gave the same response as in the previous experiments while compound **4** proved to be inactive. In order to improve the system and the solubility of the components, the concentration of all compounds was decreased proportionally while the total reaction volume was fixed to 10 μL for each dilution. For this purpose, three diluted reactions were prepared (Table A in S2 File), the inhibitor and the NF-κB protein were diluted in the same ratios for each dilution (3/4, 1/2 and 1/4), and the DNA recognition target was diluted in lower ratios (1/2, 1/4 and 1/8). Consequently, the NF-κB:DNA molar ratio was increased, while the concentration was reduced. This was done to favor the formation of the complex, nevertheless the complex demonstrated to be dissociated by the dilution (Fig. 5a). This effect becomes clear when the controls B1 (NF-κB:DNA complex without inhibitor) and B2 (free DNA recognition target in nuclease free water) are compared. The difference between B1 and B2 for the dilution I is 82% while for dilution IV it is 45%; the last dilution limited significantly the analytical range. Additionally, the resolution decreases with dilution, making it difficult to see a difference between active and inactive compounds. The second evaluated variable was the percentage of DMSO (10, 20 and 30% w/w) in the final mixture (Table B in S2 File). The results show that DMSO had a strongly negative effect on the stability of the NF-κB:DNA complex and complete dissociation was observed at 30% DMSO concentration (Fig. 5b).

### Preparation and Structure Elucidation of Damsin Derivatives

Isolation of natural products **1** and **2**, as well as the preparation of derivatives **3–10**, **12–18**, **20**, **22** and **27** (Fig. 2), was done as reported previously [30,33,34]. Semisynthesis of compounds **11**, **19**, **21** and **23–26** (Fig. 2) from **1** was also performed. Compound **11** was prepared by the ozonolysis of **5**. This derivative was intended as a 11-keto intermediate to give access to 13-substituted pseudoguaianolides via Wittig olefinations, but unfortunately **11** only exists as the enolic tautomer as shown by NMR data, that is not useful for such reactions. A hydroxylic proton was observed at low field (δ 6.24 ppm), as well as two new unsaturated carbons corresponding to C-7 (δ 135.8 ppm) and C-11 (δ 136.8 ppm).

Compound **19** was synthesized by an inversion of the configuration of C-3 of **18**, by a Mitsunobu reaction. The stereochemistry of the epimer **19** was confirmed by the correlation between H-3α and H-1α (calculated distance 2.6Å) observed in the NOESY spectrum. The coupling constants of H-3α were also compared. The experimental coupling constant with H-2α is 8.2Hz and the calculated 7.3Hz, while the experimental coupling constant with H-2β is 11.4 Hz and the calculated 9.3Hz.

**Figure 6:**
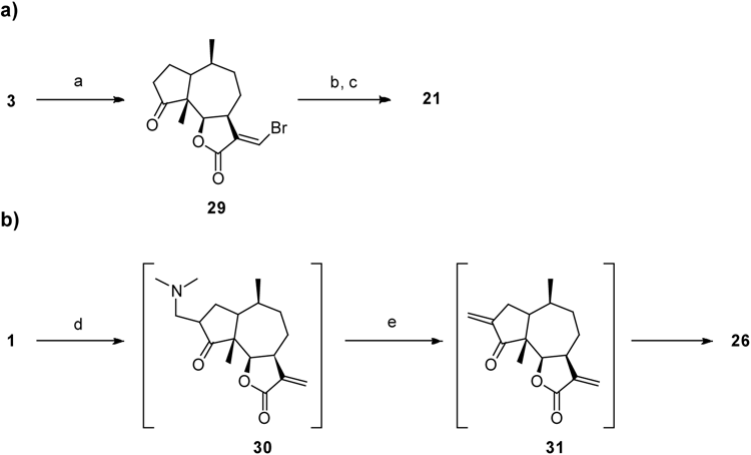
Reaction conditions. **a)** Suzuki coupling conditions: a. Et_3_N/DCM; b. PrI, Buli, B-methoxy-9-BBN/ Et_2_O, THF; c. K_3_PO_4(aq)_, **29**, PdCl_2_(dppf)⋅CH_2_Cl_2_/DMF. **b)** Mannich condensation: d. H_2_C = N^+^(CH_3_)_2_, CF_3_CO_2_-/DCM; e. MeI/MeOH.

Compound **21** was synthesized via **3** and **29** (Fig. 6a) using Suzuki coupling conditions. However, rather than obtaining the coupled product (13-propyldamsin), (*E*)-13-butyldamsin (**21**) was formed. Presumably, the mechanism is a push-pull substitution of the bromine by the *n*-butyl anion (BuLi). The configuration of the 11–13 double bond of **21** was determined by the NOESY correlations observed between H_2–_16 and H-7.

The epoxidation of the conjugated double bond in compound **9** to obtain products **23** and **24** was carried out with hydrogen peroxide in NaOH solution under microwave irradiation, but the yield of the epoxidized products was low. The configuration of the epoxide **23** was determined by the NOESY correlations between H_3–_13 and H-6, H-9α and H-1. In the same way the epimer **24** was assigned by the NOESY correlation of H_3–_13 with H-8β, which also correlates with H_3–_14 and H_3–_15. An alternative reaction with sodium hypochlorite in pyridine was tested to obtain **23** and **24**. However, instead of the desired epoxides, the 3,3-dichloro derivative **25** was obtained.

**Figure 7:**
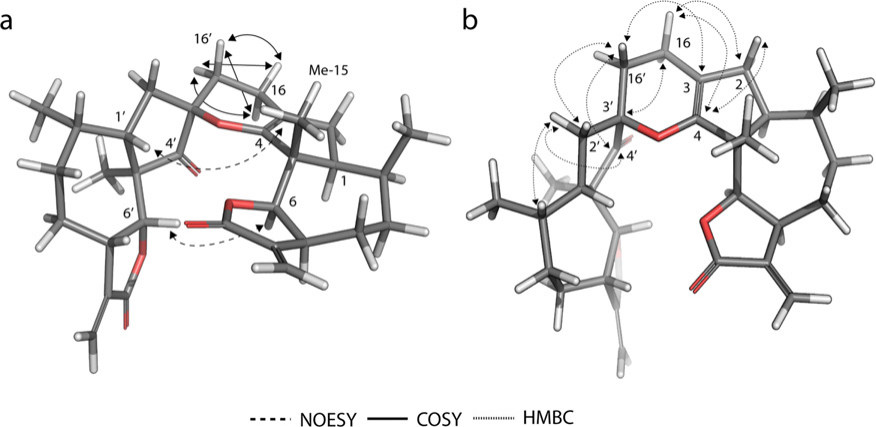
Computational model of the less energy conformer of dimer 26. The experimental 2D-NMR NOESY, COSY and HMBC correlations are indicated with arrows.

Finally, we wanted access to compound **31** (Fig. 6b) in order to have a second exocyclic Michael acceptor, however, unexpectedly it dimerized through a regioselective hetero-Diels-Alder (HDA) reaction to form **26**. The structure of dimer **26** was elucidated comparing the COSY, HMBC and NOESY spectra with computational models (Fig. 7). The regioselectivity of the reaction corresponds to an inverse-demand HDA mechanism [36], only yielding the *endo* addition adduct **26**. The COSY correlations confirmed the inverse-demand HDA, in particular the correlations between the protons on positions 16 and 16’, as well as their HMBC correlations. The *endo* addition was finally confirmed by the NOESY correlations between H-6 and H-6’ (calculated distance 2.8 Å) and between H-1’ and H_3–_15 (calculated distance 3.8 Å). The distances for the same proton pairs in the exo adduct are both greater than 6 Å.

Compound **31** was prepared via a Mannich condensation (Fig. 6b) with dimethyl(methylene)ammonium trifluoroacetate followed by a Hoffmann elimination as reported previously [35], but it could not be isolated.

### Screening of the Derivatives

**Figure 8:**
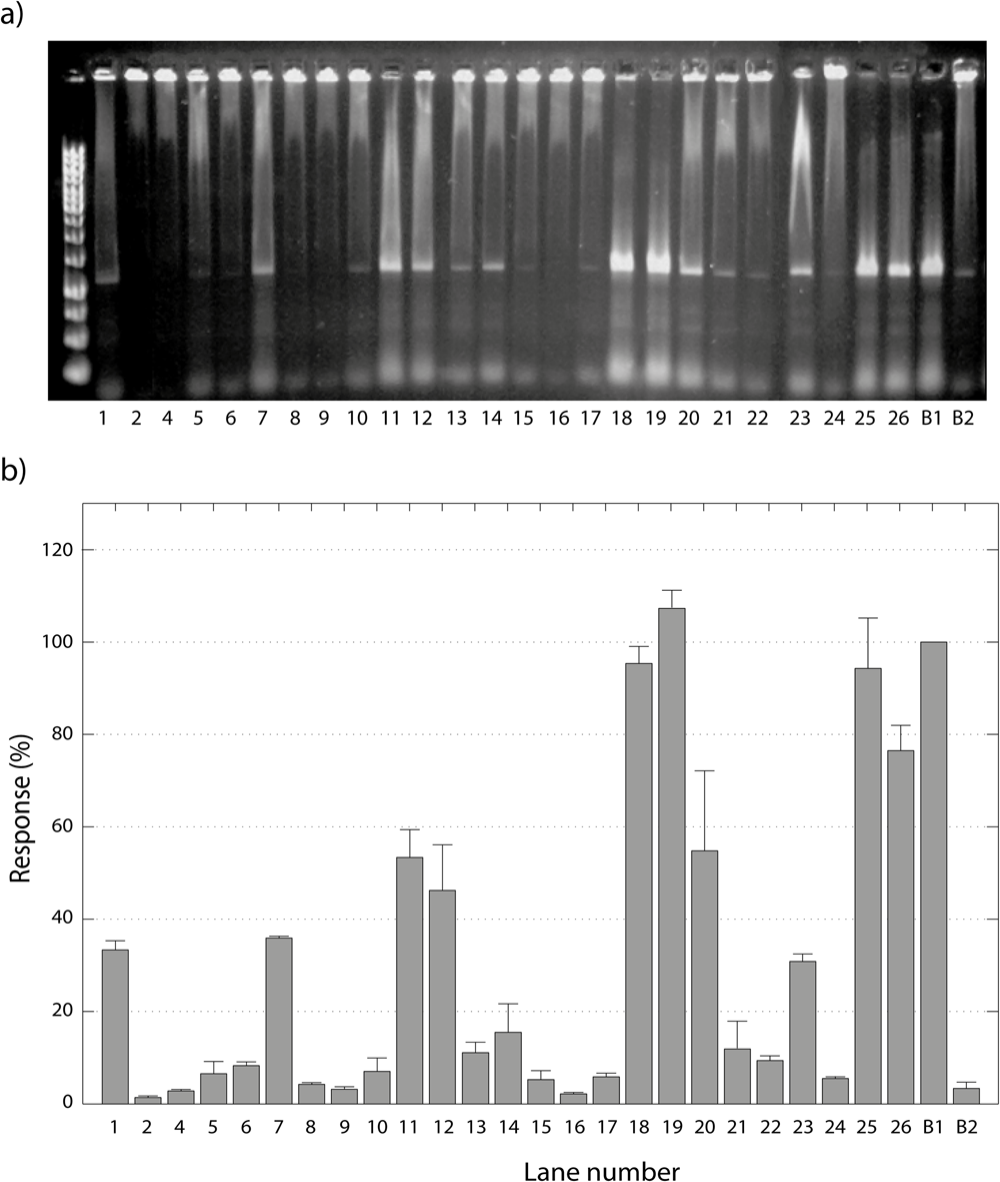
Screening of derivatives and their effect on NF-κB/DNA complex stability. **a)** Agarose gel showing the effect of the tested compounds on NF-κB:DNA complex formation. Each lane indicates the number of the compound in Fig. 2. B1 is the positive control **28**, while B2 is the negative control. **b)** Response for each compound (Fig. 2) compared to the positive control **28**. The error bars indicate the standard deviation.

The compounds tested as inhibitors of NF-κB:DNA complex formation are shown in Fig. 2. They were tested using the optimized screening conditions discussed above. *N*-Ethylmaleimide (NEM) **(28)** was used as a positive control as it is known to be a cysteine alkylating agent reacting via a Michael addition mechanism [37]. The assays were performed in quadruple replicates, and outlying data was rejected using Grubbs test [38]. All the compounds except compounds **3** and **27** showed acceptable replicability. Compound **3** is very active but is also highly electrophilic, a property that may cause it to react indistinctly with many components in the protein (e.g. cysteine and histidine residues) as well as guanine in the free DNA [39]. On the other hand, compound **27** was active but had low solubility in the HEPES buffer even with 10% DMSO. The other compounds showed satisfactory replicability with a specific response of each, facilitating a discussion of structure-activity relationships. The compounds were divided into four categories according to their responses (see Fig. 8):

**A**. Providing at least 90% of the response of the positive control. These compounds are considered to be very active, and include compounds **18**, **19** and **25**. The common characteristics of this group are a substituent at C-3, in addition to the α-methylene-γ-lactone, although how the C-3 substituent increases the activity remains unclear. A very important exception in this group is **25**, which is very active despite having the less reactive endocyclic α,β-unsaturated lactone. This indicates that the two geminal chlorines at C-3 are at least partly responsible for the high activity.

**B.** Providing between 50 and 90% of the response of the positive control. These compounds are considered to be active, and include compounds **11**, **20** and **26**. The structural diversity in this group does not allow for the identification of common or similar structural features responsible of the activity, and consequently these compounds will be discussed independently. Compound **11** is an exception among the active compounds, having features that diminish the activity (as will be described later), as a dioxalane ring in position 4 and lacks the α-methylene-γ-lactone moiety. However, the enol in the lactonic ring might be responsible for its activity. Compound **20** has two characteristics that also are present in group **A**, a substituent at C-3 and a Michael acceptor, but the lower activity suggests that the configuration of position 3 may play an important role (compared with **19**). Compound **26** has two Michael acceptors giving the possibility of cross-linking in the protein, which could explain the higher activity compared to **1**.

**C.** Providing 10 to 50% of the response of the positive control. These compounds are considered to be poorly active, and include **1**, **7**, **12**, **13**, **14**, **21** and **23**. Some compounds in this group resemble damsin **(1)**, e.g. **12** with the keto function reduced to a hydroxyl group. The fact that **12** is more active than **1** suggests that the presence of a hydroxyl group (hydrogen bond donor) in position 4, enables the molecule to interact differently with the target. Compound **14** with two Michael acceptors is nevertheless less potent than **1**. This might depend on a higher reactivity of the second Michael acceptor (the cyclopentenone) and, as a consequence, a low selectivity for the target residue (cys-38). Compound **21** is less active compared to **1**, which is logical when the steric hindrance from the butyl substitution in position 13 is considered. Three of the derivatives do not fit the previously described characteristics. Unexpectedly, compound **7** was demonstrated to be approximately as active as **1**, even if the α-methylene-γ-lactone moiety is not present. Comparing its activity with **1** and **12**, the notion that the β-hydroxyl group in position 4 is beneficial for the activity is reinforced. In the same way, compound **13** demonstrated to be slightly active even without the Michael acceptor, although it cannot be excluded that **13** spontaneously eliminates ethanol during the assay reverting to the α-methylene-γ-lactone moiety. Finally, the activity of compound **23** could be explained by the presence of an alkylating epoxide.

**D**. Less than 10% of the response of the positive control. These compounds are considered to be inactive, and include **2, 4, 5, 6, 8, 9, 10, 15, 16, 17, 22** and **24**. The main characteristics that render them inactie are: a dioxalane group in position 4 (**4** and **5**), the lack of a Michael acceptor **(4, 6, 8** and **22**), and the presence of a less reactive endocyclic α,β-unsaturated lactone **9, 15** and **17**). Other derivatives that are electrophilic but inactive are the epoxides (**16** and **24**). Compound **2** has a hydroxyl group in position 1, which somehow affects the activity negatively. Finally, **10** was designed to provide intramolecular basic catalysis for the Michael addition, is probably not chemically stable enough to survive the assay conditions.

## Conclusions

A biochemical assay based on a DNA fragment constructed with two recognition sequences for the NF-κB binding, enabling the detection of free DNA from NF-κB–DNA complex on a 1% agarose gel electrophoresis, was developed. The assay is fast, low cost and applicable for screening of potential inhibitors of the NF-κB—DNA complex formation. The assay and the following SAR study facilitated the identification of relevant structural features in the pseudoguaianolide skeleton, which are necessary for, or enhance the capacity of dissociating the complex NF-κB—DNA. The most active compounds have a substitution at C-3 (α-carbonyl), in addition to the α-methylene-γ-lactone moiety which is essential for the alkylation of RelA.

## Supporting Information

S1 File Synthesis. NMR spectra of compounds 11, 19, 21, 23, 24, 25 and 26.
Compound 11.
^1^H-NMR
^13^C-NMRCompound 19.
^1^H-NMR
^13^C-NMRNOESYCompound 21.
^1^H-NMR
^13^C-NMRNOESYCompound 23.
^1^H-NMR
^13^C-NMRNOESYCompound 24.
^1^H-NMR
^13^C-NMRNOESYCompound 25.
^1^H-NMR
^13^C-NMRCompound 26.
^1^H-NMR
^13^C-NMRNOESY
(PDF)

S2 File Supporting Information Tables. Table A. Dilutions prepared to test the effect of the concentration of the reaction components. Table B. Dilutions prepared to test the effect of DMSO concentration.
(PDF)

## References

[1] Baldwin AS (2001) Control of oncogenesis and cancer therapy resistance by the transcription factor NF-kB. J Clin Invest 107: 241–246. 10.1172/JCI11991 11160144 PMC199203

[2] Miller SC, Huang R, Sakamuru S, Shukla SJ, Attene-Ramos MS, et al (2010) Identification of known drugs that act as inhibitors of NF-κB signaling and their mechanism of action. Biochem Pharmacol 79: 1272–1280. 10.1016/j.bcp.2009.12.021 20067776 PMC2834878

[3] Kim HJ, Hawke N, Baldwin AS (2006) NF-kappaB and IKK as therapeutic targets in cancer. Cell death and differentiation, 13, 738–747. 10.1038/sj.cdd.4401877 16485028

[4] Orlowski RZ, Baldwin Jr AS (2002) NF-κB as a therapeutic target in cancer. Trends in Molecular Medicine, 8, 385–389. 10.1016/S1471-4914(02)02375-4 12127724

[5] Lin A, Karin M (2003) NF-κB in cancer: a marked target. Seminars in Cancer Biology, 13, 107–114. 10.1016/S1044-579X(02)00128-1 12654254

[6] Verma IM (2004) Nuclear factor (NF)-κB proteins: therapeutic targets. Annals of the Rheumatic Diseases, 63, ii57–ii61. 10.1136/ard.2004.028266 15479873 PMC1766777

[7] Gilmore TD (2006) Introduction to NF-[kappa]B: players, pathways, perspectives. Oncogene, 25, 6680–6684. 10.1038/sj.onc.1209954 17072321

[8] Hayden MS, Ghosh S (2008) Shared Principles in NF-κB Signaling. Cell, 132, 344–362. 10.1016/j.cell.2008.01.020 18267068

[9] Oeckinghaus A, Hayden MS, Ghosh S (2011) Crosstalk in NF-[kappa]B signaling pathways. Nat Immunol, 12, 695–708. 10.1038/ni.2065 21772278

[10] Nakanishi C, Toi M (2005) Nuclear factor-[kappa]B inhibitors as sensitizers to anticancer drugs. Nat Rev Cancer, 5, 297–309. 10.1038/nrc1588 15803156

[11] Gasparian AV, Guryanova OA, Chebotaev DV, Shishkin AA, Yemelyanov AY, et al (2009) Targeting transcription factor NFκB: comparative analysis of proteasome and IKK inhibitors. Cell Cycle, 8, 1559–1566. 10.4161/cc.8.10.8415 19372735

[12] Olivier S, Robe P, Bours V (2006) Can NF-κB be a target for novel and efficient anti-cancer agents? Biochemical Pharmacology 72: 1054–1068. 10.1016/j.bcp.2006.07.023 16973133

[13] Lai C, Jiang X, Li X (2006) Development of luciferase reporter-based cell assays. Assay and drug development technologies, 4, 307–315. 10.1089/adt.2006.4.307 16834536

[14] Israël N, Gougerot-Pocidalo MA, Aillet F, Virelizier JL (1992) Redox status of cells influences constitutive or induced NF-kappa B translocation and HIV long terminal repeat activity in human T and monocytic cell lines. The Journal of Immunology, 149, 3386–3393. 1431113

[15] Plummer SM, Holloway KA, Manson MM, Munks RJ, Kaptein A, et al (1999) Inhibition of cyclo-oxygenase 2 expression in colon cells by the chemopreventive agent curcumin involves inhibition of NF-kappaB activation via the NIK/IKK signalling complex. Oncogene, 18, 6013–6020. 10.1038/sj.onc.1202980 10557090

[16] Sun SC, Ganchi PA, Béraud C, Ballard DW, Greene WC (1994) Autoregulation of the NF-kappa B transactivator RelA (p65) by multiple cytoplasmic inhibitors containing ankyrin motifs. Proceedings of the National Academy of Sciences, 91, 1346–1350. 10.1073/pnas.91.4.1346 8108414 PMC43155

[17] Lindenmeyer MT, Garci´a-Piñeres AJ, Castro V, Merfort I (2004) Sesquiterpene lactones inhibit luciferase but not β-galactosidase activity in vitro and ex vivo. Analytical Biochemistry, 328, 147–154. 10.1016/j.ab.2004.01.021 15113690

[18] Matsusaka T, Fujikawa K, Nishio Y, Mukaida N, Matsushima K, et al (1993) Transcription factors NF-IL6 and NF-kappa B synergistically activate transcription of the inflammatory cytokines, interleukin 6 and interleukin 8. Proceedings of the National Academy of Sciences, 90, 10193–10197. 10.1073/pnas.90.21.10193 PMC47740 8234276

[19] Ma DL, Xu T, Chan DSH, Man BYW, Fong WF, et al (2011) A highly selective, label-free, homogenous luminescent switch-on probe for the detection of nanomolar transcription factor NF-kappaB. Nucleic Acids Research, 39, e67 10.1093/nar/gkr106 21398636 PMC3105395

[20] Zhao X, Vainshtein I, Gellibolian R, Shu Y, Dotimas H, et al (2003) Homogeneous assays for cellular protein degradation using beta-galactosidase complementation: NF-kappaB/IkappaB pathway signaling. Assay and drug development technologies, 1, 823–833. 10.1089/154065803772613453 15090228

[21] Ding GJF, Fischer PA, Boltz RC, Schmidt JA, Colaianne JJ, et al (1998) Characterization and Quantitation of NF-κB Nuclear Translocation Induced by Interleukin-1 and Tumor Necrosis Factor-α: development and use of a high capacity fluorescence cytometric system. Journal of Biological Chemistry, 273, 28897–28905. 10.1074/jbc.273.44.28897 9786892

[22] Finco TS, Beg AA, Baldwin AS (1994) Inducible phosphorylation of I kappa B alpha is not sufficient for its dissociation from NF-kappa B and is inhibited by protease inhibitors. Proceedings of the National Academy of Sciences, 91, 11884–11888. 10.1073/pnas.91.25.11884 PMC45340 7991551

[23] Riganti C, Doublier S, Costamagna C, Aldieri E, Pescarmona G, et al (2008) Activation of Nuclear Factor-κB Pathway by Simvastatin and RhoA Silencing Increases Doxorubicin Cytotoxicity in Human Colon Cancer HT29 Cells. Molecular Pharmacology, 74, 476–484. 10.1124/mol.108.045286 18463201

[24] Castro AC, Dang LC, Soucy F, Grenier L, Mazdiyasni H, et al (2003) Novel IKK inhibitors: β-carbolines. Bioorganic & Medicinal Chemistry Letters, 13, 2419–2422. 10.1016/S0960-894X(03)00408-6 12824047

[25] Bremner P, Heinrich M (2002) Natural products as targeted modulators of the nuclear factor-KB pathway. Journal of Pharmacy and Pharmacology, 54, 453–472. 10.1211/0022357021778637 11999122

[26] Salminen A, Lehtonen M, Suuronen T, Kaarniranta K, Huuskonen J (2008) Terpenoids: natural inhibitors of NF-κB signaling with anti-inflammatory and anticancer potential. Cell. Mol. Life Sci., 65, 2979–2999. 10.1007/s00018-008-8103-5 18516495 PMC11131807

[27] Rüngeler P, Castro V, Mora G, Gören N, Vichnewski W, et al (1999) Inhibition of transcription factor NF-κB by sesquiterpene lactones: a proposed molecular mechanism of action. Bioorganic & Medicinal Chemistry, 7, 2343–2352. 10.1016/S0968-0896(99)00195-9 10632044

[28] Garcia-Pineres AJ, Castro V, Mora G, Schmidt TJ, Strunck E, et al (2001) Cysteine 38 in p65/NF-κB plays a crucial role in DNA binding inhibition by sesquiterpene lactones. J. Biol. Chem., 276, 39713–39720. 10.1074/jbc.M101985200 11500489

[29] Johansson MH (2012) Reversible Michael additions: covalent inhibitors and prodrugs. Mini Rev Med Chem, 12, 1330–1344. 10.2174/13895575112091330 22625413

[30] Villagomez R, Rodrigo GC, Collado IG, Calzado MA, Muñoz E, et al (2013) Multiple Anticancer Effects of Damsin and Coronopilin Isolated from Ambrosia arborescens on Cell Cultures. Anticancer Research, 33, 3799–3805. 24023312

[31] Chen FE, Huang DB, Chen YQ, Ghosh G (1998) Crystal structure of p50/p65 heterodimer of transcription factor NF-[kappa]B bound to DNA. Nature, 391, 410–413. 10.1038/34956 9450761

[32] Di L, Kerns EH (2006) Biological assay challenges from compound solubility: strategies for bioassay optimization. Drug Discovery Today, 11, 446–451. 10.1016/j.drudis.2006.03.004 16635808

[33] Villagomez R, Collado JA, Muñoz E, Almanza G, Sterner O (2014) Natural and semi-synthetic pseudoguaianolides as inhibitors of NF-κB. Journal of Biomedical Science and Engineering. 7, 833–847. 10.4236/jbise.2014.710083

[34] Villagomez R (2014) Biological activities of natural and semi-synthetic pseudo-guaianolides: Inhibition of transcription factors, Doctoral Thesis, Lund University, Lund Sweden.

[35] Ahond A, Cave A, Kan-Fan C, Husson HP, De Rostolan J, et al (1968) Facile N-O bond cleavages of amine oxides. Journal of the American Chemical Society, 90, 5622–5623. 10.1021/ja01022a063

[36] Ding C, Wang L, Chen H, Wild C, Ye N, et al (2014) ent-Kaurane-based regio- and stereoselective inverse electron demand hetero-Diels–Alder reactions: synthesis of dihydropyran-fused diterpenoids. Organic & Biomolecular Chemistry, 12, 8442–8452. 10.1039/C4OB01040J 25225052 PMC4192081

[37] Garcia-Pineres AJ, Lindenmeyer MT, Merfort I (2004) Role of cysteine residues of p65/NF-κB on the inhibition by the sesquiterpene lactone parthenolide and N-ethyl maleimide, and on its transactivating potential. Life Sci., 75, 841–856. 10.1016/j.lfs.2004.01.024 15183076

[38] Grubbs FE (1969) Procedures for detecting outlying observations in samples. Technometrics, 11, 1–21. 10.1080/00401706.1969.10490657

[39] Djalali-Behzad G, Hussain S, Osterman-Golkar S, Segerbäck D (1981) Estimation of genetic risks of alkylating agents: VI. Exposure of mice and bacteria to methyl bromide. Mutation Research/Fundamental and Molecular Mechanisms of Mutagenesis, 84, 1–9. 10.1016/0027-5107(81)90044-0 7035924

[40] Chen FE, Kempiak S, Huang DB, Phelps C, Ghosh G (1999) Construction, expression, purification and functional analysis of recombinant NFκB p50/p65 heterodimer. Protein Engineering, 12, 423–428. 10.1093/protein/12.5.423 10360983

[41] Sue SC, Cervantes C, Komives EA, Dyson HJ (2008) Transfer of flexibility between ankyrin repeats in IκBα upon formation of the NF-κB complex. Journal of Molecular Biology, 380, 917–931. 10.1016/j.jmb.2008.05.048 18565540 PMC2603615

