# Supplementary material for: Effect of Natural and Semisynthetic Pseudoguianolides on the Stability of NF-κB:DNA Complex Studied by Agarose Gel Electrophoresis
Source: PLoS One. 2015 Jan 23;10(1):e0115819. doi: 10.1371/journal.pone.0115819 (PMC4304792; doi:10.1371/journal.pone.0115819)

Effect of Natural and Semisynthetic Pseudoguianolides on the Stability of NF-κB:DNA Complex Studied by Agarose Gel Electrophoresis

Rodrigo Villagomez, Rajni Hatti-Kaul, Olov Sterner, Giovanna Almanza, Javier A. Linares-Pastén

Supporting Information

S1 Synthesis

(6S,9aR,9bR)-3-hydroxy-6,9a-dimethyl-4,5,6,6a,7,8,9a,9b-octahydro-2H-spiro[azuleno[4,5-b]furan-9,2'-[1,3]dioxolan]-2-one (11). To a cold solution (-78 °C) of compound **5** (462.6 mg, 1.583 mmol) in dry DCM (30 mL), ozone was bubbled continuously for 1 h, until the solution became light blue. Then, the reaction mixture was quenched by addition of Me_2S (347.2 μL, 4.750 mmol) at -78 °C and the reaction mixture was stirred at the same temperature for 3.5 h. The volatile components were removed *in vacuo* and the crude product was purified by silica gel flash chromatography (30 % EtOAc:Pet.Et.$_{40-60}$) yielding **11** (318.3 mg, 68 %). $[\alpha]_D^{20}$: –33.4 (*c* 1.00, CH_2Cl_2); IR (film) = 3361, 2960, 2879, 1735, 1459, 1386, 1264, 1175, 1134, 1082, 1056, 1018, 970, 952, 882, 774, 734, 661, 599, 559, 534, 518, 465 cm^{-1}. (400 MHz $CDCl_3$) δ 2.23 (1H, *ddd*, ^3J 12.1; 7.9; 4.5Hz, H-1), δ 1.60 (1H, *m*, H-2a), δ 1.76 (1H, *m*, H-2b), δ 1.82 (1H, *m*, H-3a), δ 1.93 (1H, *ddd*, ^3J 13.5; 4.3; 2.2Hz, H-3b), δ 5.16 (1H, *t*, ^3J 1.7; 1.7 Hz, H-6), δ 2.58 (1H, *dddd*, ^3J 19.2; 5.2; 3.6; 1.3 Hz,H-8a), δ 2.72 (1H, *dddd*, ^3J 19.2; 11.5; 4.5; 2.1 Hz, H-8b), δ 1.75 (1H, *m*, H-9a), δ 1.75 (1H, *m*, H-9b), δ 2.09 (1H, *m*, H-10), δ 6.24 (1H, *bs*, C-OH), δ 0.96 (3H, *d*, ^3J 7.5 Hz, Me-14), δ 0.77 (3H, *s*, Me-15), δ 3.88 (2H, *m*, H-16), δ 4.07 (2H, *m*, H-17); d_C (100.6 MHz, $CDCl_3$) 48.1 (C-1), 23.2 (C-2), 34.6 (C-3), 119.1 (C-4), 52.1 (C-5), 83.4 (C-6), 135.8 (C-7), 19.5 (C-8), 31.3 (C-9), 33.4 (C-10), 136.8 (C-11), 170.6 (C12), 13.6 (C-14), 10.9 (C-15), 65.0 (C-16), 64.8 (C-17); HRMS-ESI: [$M+H^+$], found 295.1571. $C_{16}H_{23}O_5$ requires 295.1545.

3β-acetoxydamsin (19) To a mixture of **18** (95.1 mg, 0.360 mmol), PPh_3 (141.5 mg, 0.540 mmol), AcOH (31 μl, 0.540 mmol), in dry DCM (7 ml) at 0 °C, DIAD (113.8 μl, 0.540 mmol) was added dropwise. The reaction was allowed to reach room temperature and was stirred for 23 h. After that time, the reaction mixture was poured into water (20 ml) and the aqueous mixture was extracted with DCM (3 x 20 ml). The combined organic layers were dried with Na_2SO_4 and concentrated *in vacuo* to give the crude product, which was purified by silica gel flash chromatography (15 % EtOAc:Pet.Et.$_{40-60}$) to yield the product **19** (19.8 mg, 18 %). $[\alpha]_D^{20}$: –14 (*c*1.00, CH_2Cl_2); IR (film) = 3434, 2962, 2933, 2879, 1740, 1459, 1373, 1230, 1185, 1088, 1045, 1022, 974, 803, 733, 701 cm^{-1}; (400 MHz $CDCl_3$) δ 2.11 (1H, *m*, H-1),δ 1.94 (1H, *m*, H-2a),δ 2.26 (1H, *m*, H-2b),δ 5.20 (1H, *dd*, ^3J 11.4; 8.2Hz, H-3a),δ 4.46 (1H, *d*, ^3J 8.7 Hz, H6),δ 3.35 (1H, *m*, H-7),δ 1.92 (1H, *m*, H-8a),δ 2.04 (1H, *m*, H-8b),δ 1.77 (1H, *m*, H-9a),δ 1.77 (1H, *m*, H-9b),δ 2.21 (1H, *m*, H-10),δ 5.51 (1H, *d*, ^3J 3.1 Hz, H-13a),δ 6.26 (1H, *d*, ^3J 3.4 Hz, H-13b),δ 1.03 (3H, *d*, ^3J 7.5 Hz, Me-14),δ 1.12 (3H, *s*, Me-15),δ 2.11 (3H, *s*, H-17); d_C (100.6 MHz, $CDCl_3$) 39.7, 30.5, 74.6, 211.5, 53.5, 81.1, 43.9, 24.8, 32.1, 33.7, 138.7, 170.1, 120.6, 16.0, 14.9, 170.0, 20.7; HRMS-ESI: [$M+H^+$], found 307.1557. $C_{17}H_{22}O_5$ requires 307.1545.

(E)-13-n-Butildamsin (21). To a solution of propyl iodide (16.0 µL, 0.162 mmol) in Et_2O (0.6 mL) at -78 °C, BuLi (203.0 µL, 0.325 mmol, 1.6 M in hexanes) was added dropwise. Immediately, a dilution of B-methoxy-9-BBN (375.0 µL, 0.375 mmol, 1.0 M in hexane) in 0.6 mL of THF was added dropwise. The reaction was allowed to reach room temperature and was stirred for 2 h, then a solution of $K_3PO_{4(aq)}$ (125.0 µL, 0.375 mmol, 3 M) followed by a solution of **29** (40.9 mg, 0.125 mmol) in DMF (0.85 mL) and the solid catalyst [$PdCl_2$(dppf).CH_2Cl_2] (5.1 mg, 0.006 mmol, 5 % mol). The reaction was stirred at room temperature and covered from light for 3 days, after which it was quenched with 10 mL of water and the aqueous layer was extracted with Et_2O (3x10 mL). The pooled organic layers were dried with Na_2SO_4 and concentrated *in vacuo*. The product was purified by silica gel flash chromatography (from 10% to 40% EtOAc:Pet.Et.$_{40\text{-}60}$) yielding **21** (11.8 mg, 31 %). $[\alpha]_D^{20}$: +2.0 (*c* 1.00, CH_2Cl_2); IR (film) = 2957, 2927, 2861, 1739, 1673, 1451, 1385, 1331, 1248, 1221, 1193, 1155, 1130, 1051, 1015, 987, 948, 886, 844, 799, 729, 631, 535 cm^{-1}. (400 MHz $CDCl_3$) δ 2.05 (1H, *m*, H-1), δ 1.84 (1H, *m*, H-2a), δ 2.07 (1H, *m*, H-2b), δ 2.20 (1H, *m*, H-3a), δ 2.47 (1H, *m*, H-3b), δ 4.43 (1H, *d*, ^3J 7.4 Hz, H6), δ 3.11 (1H, *m*, H-7), δ 1.49 (1H, *m*, H-8a), δ 1.94 (1H, *m*, H-8b), δ 1.71 (1H, *m*, H-9a), δ 1.85 (1H, *m*, H-9b), δ 2.22 (1H, *m*, H-10), δ 6.67 (1H, *ddd*, ^3J 8.3; 7.3; 2.1 Hz, H-13a), δ 1.09 (3H, *d*, ^3J 7.6 Hz, Me-14), δ 1.19 (3H, *s*, Me-15), δ 2.18 (2H, *m*, H-16), δ 1.45 (2H, *m*, H-17), δ 1.34 (2H, *m*, H-18), δ 0.90 (3H, *t*, ^3J 7.2 Hz, H-19); d_C (100.6 MHz, $CDCl_3$) 46.0 (C-1), 24.3 (C-2), 35.1 (C-3), 220.5 (C-4), 54.8 (C-5), 80.6 (C-6), 45.0 (C-7), 25.9 (C_8), 36.7 (C-9), 34.8 (C-10), 131.8 (C-11), 170.7 (C-12), 141.0 (C-13), 16.2 (C-14), 15.2 (C-15), 29.2 (C-16), 30.6 (C-17), 22.4 (C-18), 13.8 (C-19); HRMS-ESI: [M+H$^+$], found 305.2142. $C_{19}H_{28}O_3$ requires 305.2117.

(7*R*, 11*R*)-Epoxy-13-hydrodamsin and (7*S*, 11*S*)-Epoxy-13-hydrodamsin (23 and 24). A mixture of **9** (50 mg, 0.201 mmol), 30 % H_2O_2 (205 µL, 2.013 mmol) and 3M NaOH solution (68 µL, 0.201 mmol) in MeOH (2 mL), was microwave irradiated at 40 °C. for 39 h. After that time a second addition of 30 % H_2O_2 (205 µL, 2.013 mmol) and 3M NaOH solution (68 µL, 0.201 mmol) in MeOH (2 mL), was done and it was irradiated at 40 °C for 4 h. The reaction was quenched by adding 1 M HCl (pH=1), and then neutralized with saturated $NaHCO_3$ and the aqueous mixture was extracted with DCM (4x10 mL). The pooled organic layers were dried with Na_2SO_4 and concentrated *in vacuo* to give the crude product, which was purified by silica gel flash chromatography (18 % EtOAc:Pet.Et.$_{40\text{-}60}$) yielding **23** (17.5 mg, 33 %). $[\alpha]_D^{20}$: +290 (*c* 1.00, CH_2Cl_2); IR (film) = 2974, 2946, 2925, 2884, 1773, 1736, 1459, 1451, 1382, 1346, 1321, 1289, 1159, 1112, 1068, 1048, 1024, 1003, 994, 963, 953, 875, 840, 719, 701, 651, 593, 542, 463, 440 cm^{-1}; (400 MHz $CDCl_3$) δ 2.07 (1H, *m*, H-1), δ 1.85 (1H, *m*, H-2a), δ 1.85 (1H, *m*, H-2b), δ 2.20 (1H, *m*, H-3a), δ 2.39 (1H, *m*, H-3b), δ 4.57 (1H, *s*, H-6), δ 1.57 (1H, *m*, H-8a), δ 2.53 (1H, *m*, H-8b), δ 1.75 (1H, *m*, H-9a), δ 1.86 (1H, *m*, H-9b), δ 2.27 (1H, *m*, H-10), δ 1.57 (3H, *s*, H-13a), δ 1.10 (3H, *d*, ^3J 7.6 Hz, Me-14), δ 1.26 (3H, *s*, Me-15); d_C (100.6 MHz, CDCl3) 44.2 (C-1), 24.9 (C-2), 38.0 (C-3), 216.7 (C-4), 52.4 (C-5), 85.8 (C-6), 72.4 (C-7), 24.1 (C-8), 33.5 (C-9), 33.9 (C-10), 61.3 (C-11), 172.0 (C-12), 11.0 (C-13), 16.2 (C-14), 20.8 (C-15); HRMS-ESI: [M+H$^+$], found 265.1459. $C_{15}H_{20}O_4$ requires 265.1440. **24** (2.9 mg, 5 %). $[\alpha]_D^{20}$: +3.0 (*c* 1.00, CH_2Cl_2); IR (film) = 2960, 2929, 2880, 1773, 1738, 1461, 1381, 1351, 1321, 1293, 1163, 1105, 1066, 1002, 961, 889, 847, 802, 746, 646, 574, 556, 541, 451 cm^{-1}; (400 MHz $CDCl_3$) δ 2.30 (1H, *m*, H-1), δ 1.94 (1H, *m*, H-2a), δ 1.94 (1H, *m*, H-2b), δ 2.29 (1H, *m*, H-3a), δ 2.47 (1H, *m*, H-3b), δ 4.32 (1H, *s*, H6), δ 1.80 (1H, *dd*, ^{3}J 15.5; 8.4 Hz, H-8a), δ 2.18 (1H, *m*, H-8b), δ 1.49 (1H, *m*, H-9a), δ 2.07 (1H, *m*, H-9b), δ 2.21 (1H, *m*, H-10), δ 1.54 (3H, *s*, H-13a), δ 1.07

(3H, *d*, ^{3}J 7.2 Hz, Me-14), δ 1.01 (3H, *s*, Me-15); d_C (100.6 MHz, $CDCl_3$) 43.4 (C-1), 23.7 (C-2), 37.2 (C-3), 217.2 (C-4), 52.1 (C-5), 83.3 (C-6), 69.5 (C-7), 24.8 (C-8), 30.3 (C-9), 33.1 (C-10), 60.2 (C-11), 172.9 (C-12), 9.0 (C-13), 17.4 (C-14), 13.0 (C-15); HRMS-ESI: [$M+H^+$], found 265.1411. $C_{15}H_{20}O_4$ requires 265.1440.

3,3-Dichlorodamsin (25). To a solution of **9** (50 mg, 0.201 mmol) in pyridine (1 mL), 250 µL of $NaClO_{aq}$ (0.503 mmol, 13 % of Cl_2), were added at 0 °C and the mixture was stirred for 17.5 h at 0 °C. The reaction was quenched by adding brine (10 mL) and the aqueous mixture was extracted with DCM (3x20 mL). The pooled organic layers were dried with Na_2SO_4, concentrated *in vacuo* to give the crude product, which was purified by silica gel flash chromatography (25 % EtOAc:Pet.Et.$_{40\text{-}60}$) yielding **25** (14.3 mg, 22 %). $[\alpha]_{\mathbf{D}}^{20}$: –65.2 (*c* 1.00, CH_2Cl_2); IR (film) = 2963, 2929, 2875, 1752, 1670, 1452, 1383, 1298, 1264, 1214, 1181, 1093, 1057, 1029, 1008, 965, 868, 826, 800, 780, 754, 730, 695, 641, 605, 539, 477 cm^{-1};(400 MHz $CDCl_3$) δ 2.75 (1H, *m*, H-1), δ 2.60 (1H, *t*, ^3J 14.8Hz, H-2a), δ 2.75 (1H, *m*, H-2b), δ 4.73 (1H, *s*, H6), δ 2.31 (1H, *m*, H-8a), δ 2.84 (1H, *dd*, ^3J 14.4; 7.2 Hz, H-8b), δ 1.28 (1H, *m*, H-9a), δ 2.22 (1H, *m*, H-9b), δ 2.29 (1H, *m*, H-10), δ 1.85 (3H, *d*, ^3J 0.7 Hz, H-13a), δ 1.00 (3H, *d*, ^3J 7.0 Hz, Me-14), δ 0.97 (3H, *s*, Me-15); d_C (100.6 MHz, $CDCl_3$) 38.1 (C-1), 45.6 (C-2), 83.2 (C-3), 201.7 (C-4), 50.1 (C-5), 85.6 (C-6), 159.6 (C-7), 23.5 (C-8), 32.3 (C-9), 32.1 (C-10), 125.1 (C-11), 174.0 (C-12), 8.4 (C-13), 17.6 (C-14), 13.1 (C-15); HRMS-ESI: [$M+H^+$], found 317.0728. $C_{15}H_{18}Cl_2O_3$ requires 317.0711.

(3aS,3a'S,6S,6'S,8S,9aR,9bR,11b'R,11c'R)-6,6',9a,11b'-tetramethyl-3,3'-dimethylene-3a,3a',4,4',5,5',6,6a,6',6a',7,7',8',9'-tetradecahydro-2H-spiro[azuleno[4,5-b]furan-8,10'-furo[3',2':7,8]azuleno[1,2-b]pyran]-2,2',9(3H,3'H,9aH,9bH,11b'H,11c'H)-trione (26). Preparation of dimethyl(methylene)ammonium trifluoroacetate: Triflouroacetic anhydride (123.2 µL, 0.886 mmol) was added dropwise to a stirred solution of trimethylamine N-oxide (66.4 mg, 0.884 mmol) in dry DCM (4.4 mL) at 0 °C. The mixture was left at room temperature for 1 h and quenched via evaporation *in vacuo* at 100 °C. The residual crude oil was disolved in DCM (5 mL) and compound **1** (100 mg, 0.403 mmol) was added as a solution in DCM (10 mL). The solution was refluxed for 37 h after which the volatile components were removed *in vacuo*. The crude material was redissolved in MeOH (4 mL), and MeI (50 µL, 0.803 mmol) was added, and the mixture was stirred at room temperature for 1h. Finally, the reaction was quenched adding saturated $NaHCO_{3(aq)}$ (20 mL) and the aqueous mixture was extracted with DCM (3x20 mL). The pooled organic layers were dried over Na_2SO_4 to give the crude product, which was purified by silica gel flash chromatography (20 % EtOAc:Pet.Et.$_{40\text{-}60}$) yielding **26** (74.2 mg, 71 %). $[\alpha]_{\mathbf{D}}^{20}$: –8.1 (*c* 1.00, CH_2Cl_2); IR (film) = 2951, 2916, 2868, 2837, 1752, 1340, 1318, 1269, 1241, 1204, 1156, 1114, 1082, 1020, 1001, 975, 940, 910, 887, 814, 732, 699, 643, 399 cm^{-1}; (500 MHz $CDCl_3$) δ 2.11 (1H, *m*, H-1), δ 1.93 (1H, *m*, H-2a), δ 2.32 (1H, *m*, H-2b), δ 4.39 (1H, *d*, ^3J 8.9 Hz, H-6), δ 3.27 (1H, *m*, H-7), δ 1.87 (1H, *m*, H-8a), δ 2.01 (1H, *m*, H-8b), δ 1.67 (1H, *m*, H-9a), δ 1.75 (1H, *m*, H-9b), δ 2.09 (1H, *m*, H-10), δ 5.45 (1H, *d*, ^3J 3.2 Hz, H-13a), δ 6.18 (1H, *d*, ^3J 3.4 Hz, H-13b), δ 1.05 (3H, *d*, ^3J 6.8 Hz, Me-14), δ 1.05 (3H, *s*, Me-15), δ 1.89 (1H, *m*, H-16a),δ 2.38 (1H, *m*, H-16b); δ 2.81 (1H, *dt*, ^3J 13.3; 5.3; 5.3Hz, H-1'), δ 1.69 (1H, *m*, H-2'a), δ 1.85 (1H, *m*, H-2'b), δ 4.72 (1H, *d*, ^3J 8.6 Hz, H-6'), δ 3.45 (1H, *m*, H-7'), δ 2.01 (1H, *m*, H-8'a), δ 2.07 (1H, *m*, H-8'b), δ 1.68 (1H, *m*, H-9'a), δ 1.82 (1H, *m*, H-'b), δ 2.13 (1H, *m*, H-10'), δ 5.45 (1H, *d*, ^3J 3.3 Hz, H-13'a), δ 6.21 (1H, *d*, ^3J 3.4 Hz, H-13'b), δ 0.98 (3H, *d*, ^3J 7.4 Hz, Me-14'), δ 1.05 (3H, *s*, Me-15'), δ 1.63 (1H, *m*, H-16'a),δ

1.87 (1H, *m*, H-16'b); d_C (125 MHz, $CDCl_3$) 45.4 (C-1), 33.7 (C-2), 105.7 (C-3), 153.2 (C-4), 52.1 (C-5), 85.5 (C-6), 43.9 (C-7), 25.4 (C-8), 33.0 (C-9), 33.7 (C-10), 139.9 (C-11), 170.7 (C-12), 119.9 (C-13), 15.9 (C-14), 15.5 (C-15), 19.0 (C-16), 38.8 (C-1'), 37.2 (C-2'), 79.9 (C-3'), 212.4 (C-4'), 54.1 (C-5'), 82.1 (C-6'), 44.1 (C-7'), 24.6 (C-8'), 31.3 (C-9'), 33.5 (C-10'), 139.1 (C-11'), 170.7 (C-12'), 119.6 (C-13'), 16.1 (C-14'), 15.0 (C-15'), 25.8 (C-16'); HRMS-ESI: [$M+H^+$], found 521.2932. $C_{32}H_{40}O_6$ requires 521.2903.

(E)-13-Bromodamsin (29). To a solution of **3** (66.8 mg, 0.164 mmol) in DMF (0.7 mL), Et_3N (25 μL, 0.180 mmol) was added and the mixture was stirred at room temperature for 22 h. The reaction was quenched with 4 ml of HCl sol. (1 M) and 10 ml of water. The aqueous mixture was extracted with DCM (3x15ml). The pooled organic layers were dried over Na_2SO_4 and concentrated *in vacuo* to give the crude product, which was purified by silica gel flash chromatography (18 % EtOAc:Pet.Et.$_{40-60}$) yielding **29** (40.9 mg, 76 %). $[\alpha]_D^{20}$: –29.0 (*c* 1.00, CH_2Cl_2); IR (film) = 2942, 2883, 2870, 1742, 1623, 1477, 1334, 1280, 1186, 1158, 1131, 1051, 1015, 988, 771, 719, 631, 572, 533 cm^{-1}; (400 MHz $CDCl_3$) δ 2.04 (1H, m, H-1), δ 1.85 (1H, m, H-2a), δ 2.05 (1H, m, H-2b), δ 2.21 (1H, m, H-3a), δ 2.47 (1H, m, H-3b), δ 4.50 (1H, d, ^3J 7.3 Hz, H6), δ 3.20 (1H, m, H-7), δ 1.86 (1H, m, H-8a), δ 1.90 (1H, m, H-8b), δ 1.72 (1H, m, H-9a), δ 1.87 (1H, m, H-9b), δ 2.22 (1H, m, H-10), δ 7.47 (1H, d, ^3J 2.2 Hz, H-13a), δ 1.09 (3H, d, ^3J 7.6 Hz, Me-14), δ 1.19 (3H, s, Me-15); d_C (100.6 MHz, CDCl3) 45.8 (C-1), 24.2 (C-2), 35.0 (C-3), 220.2 (C-4), 54.7 (C-5), 80.7 (C-6), 47.6 (C-7), 23.2 (C-8), 36.4 (C-9), 34.6 (C-10), 137.9 (C-11), 167.0 (C-12), 119.8 (C-13), 16.2 (C-14), 15.0 (C-15); HRMS-ESI: , found 349.0417, $C_{15}H_{19}BrO_3$ requires 349.0415.

NMR spectra of compounds 11, 19, 21 23, 24, 25 and 26.

^{1}H-NMR

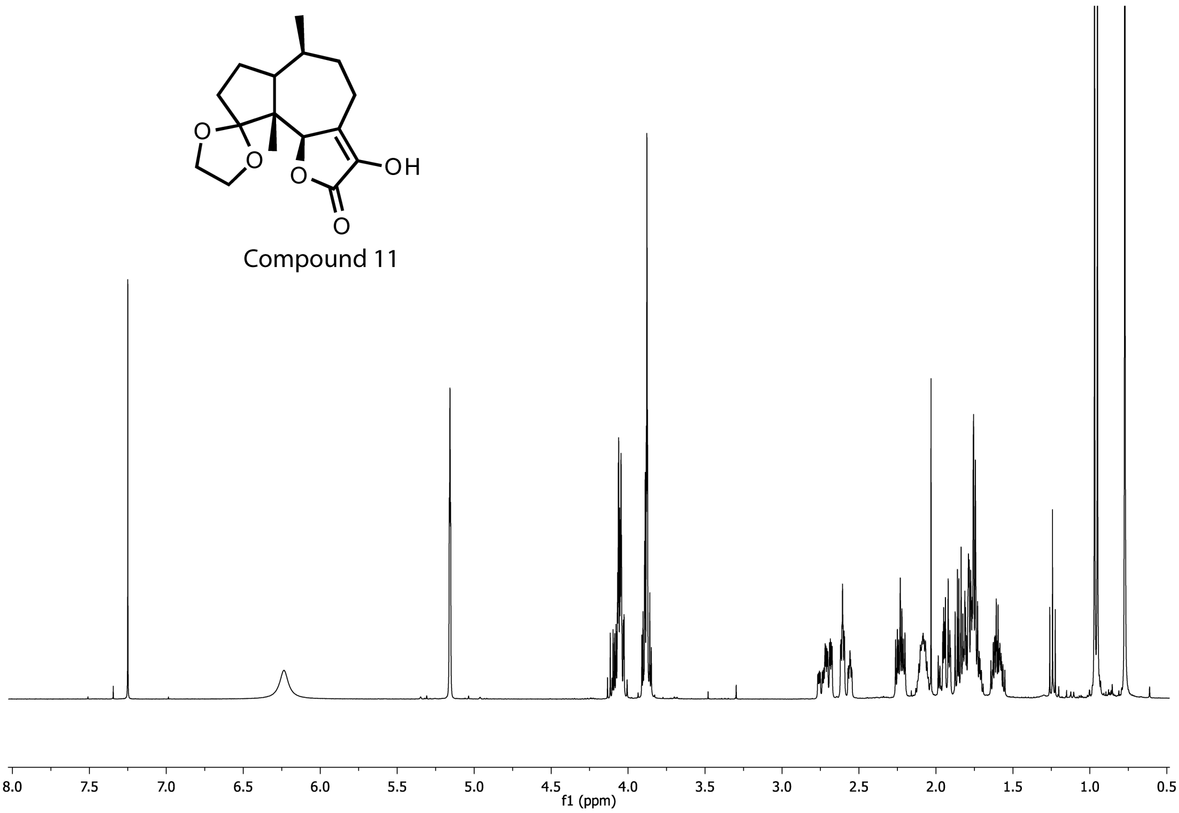

^{13}C-NMR

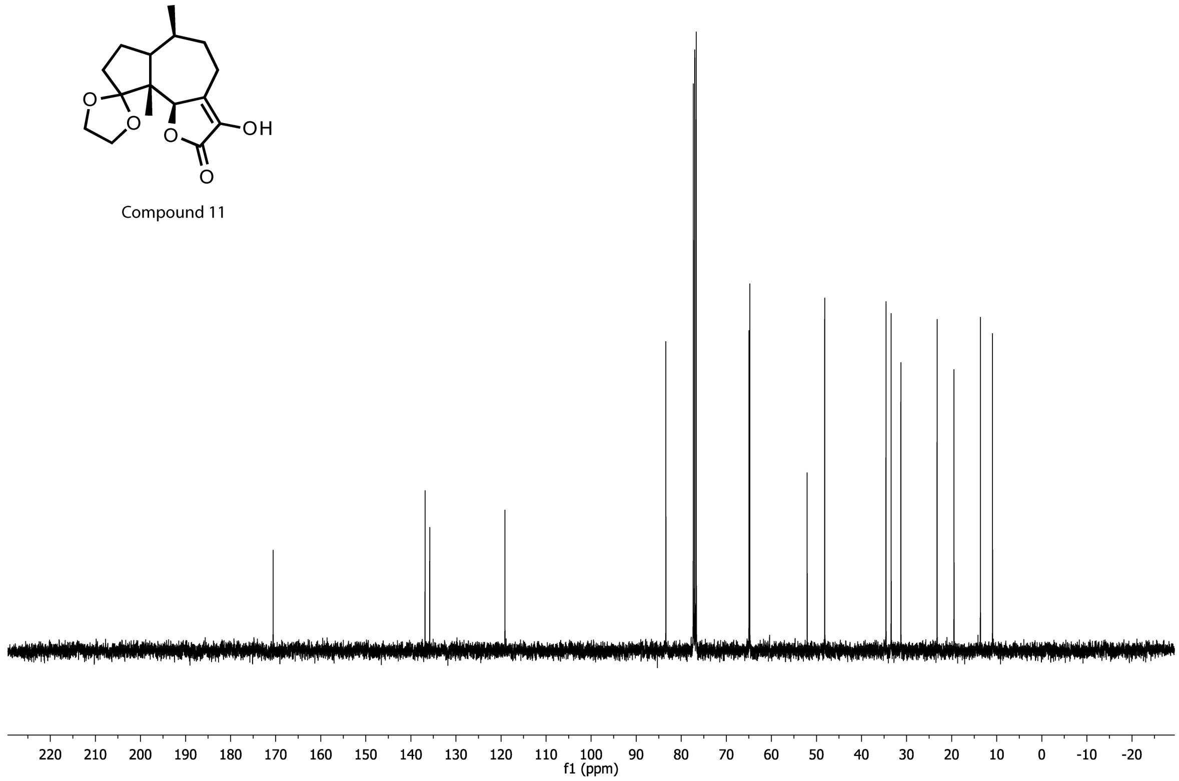

^{1}H-NMR

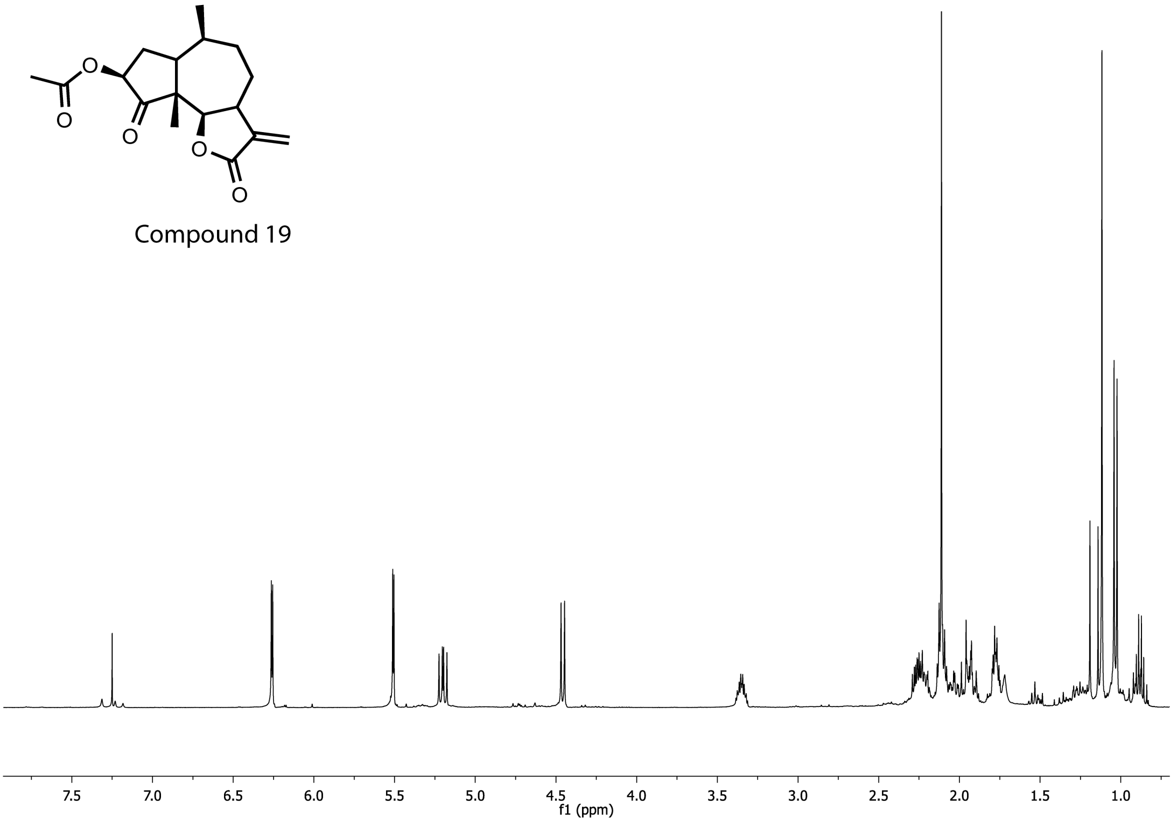

^{13}C-NMR

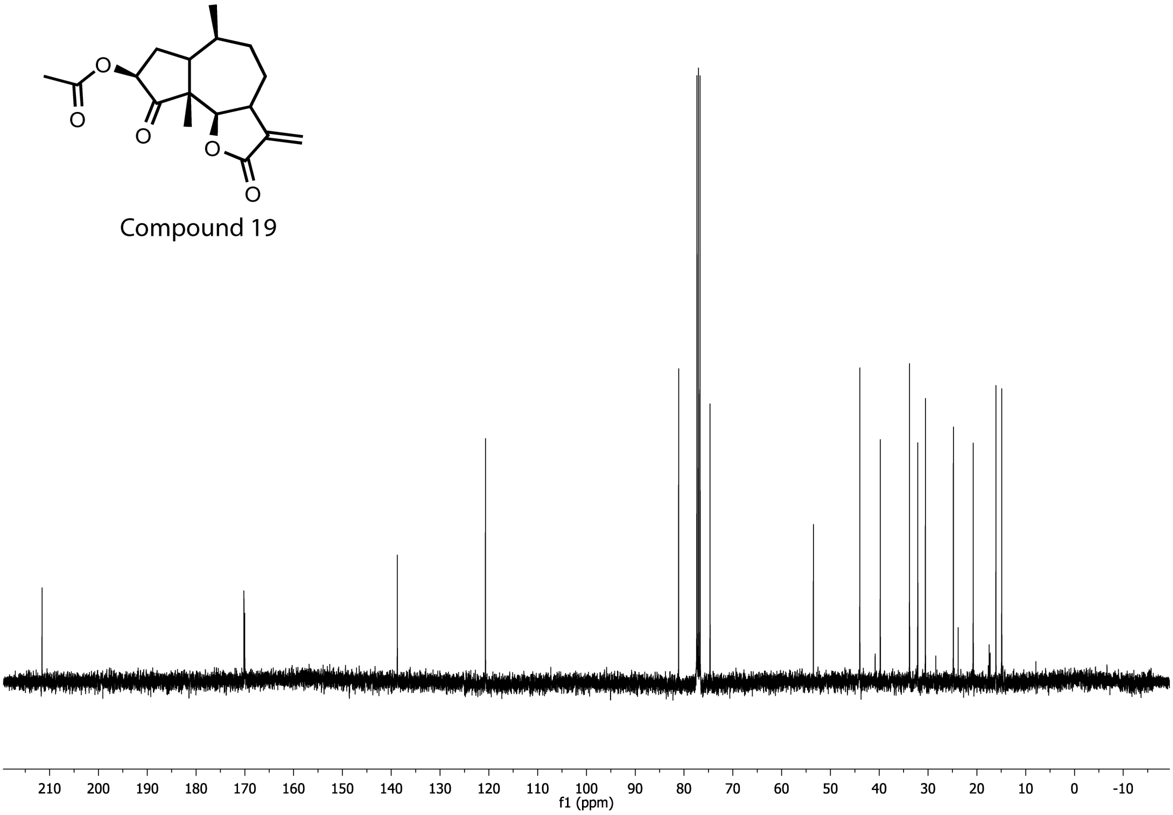

NOESY

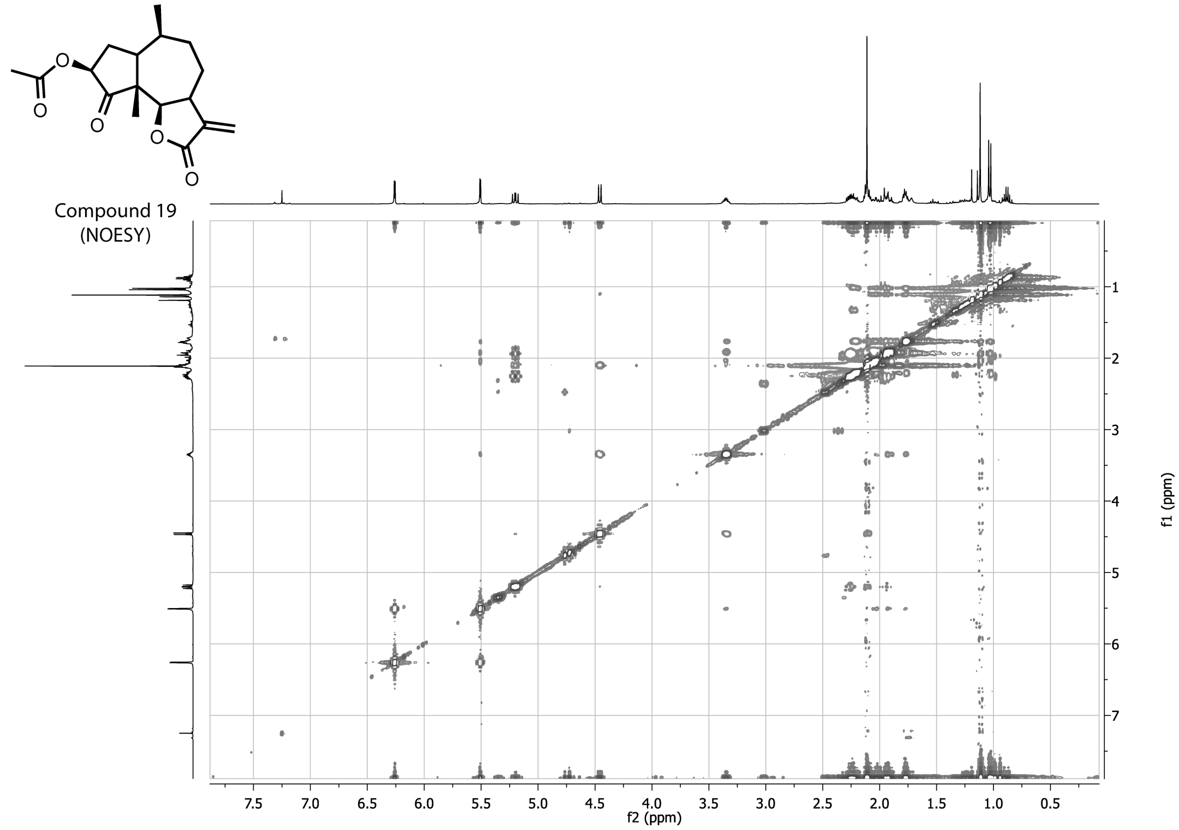
Compound 19
(NOESY)
f2 (ppm)
f1 (ppm)
7.5 7.0 6.5 6.0 5.5 5.0 4.5 4.0 3.5 3.0 2.5 2.0 1.5 1.0 0.5
1 2 3 4 5 6 7

^{1}H-NMR

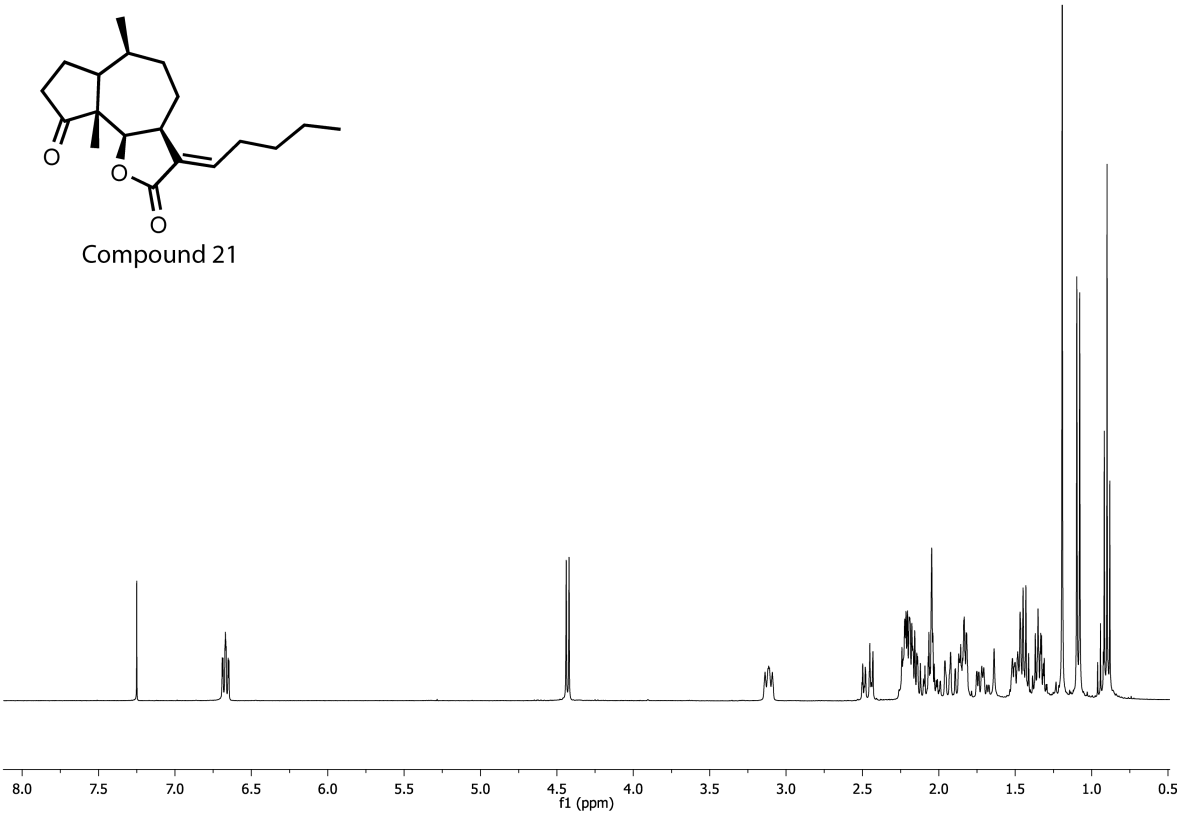
Compound 21
f1 (ppm)
8.0 7.5 7.0 6.5 6.0 5.5 5.0 4.5 4.0 3.5 3.0 2.5 2.0 1.5 1.0 0.5

^{13}C-NMR

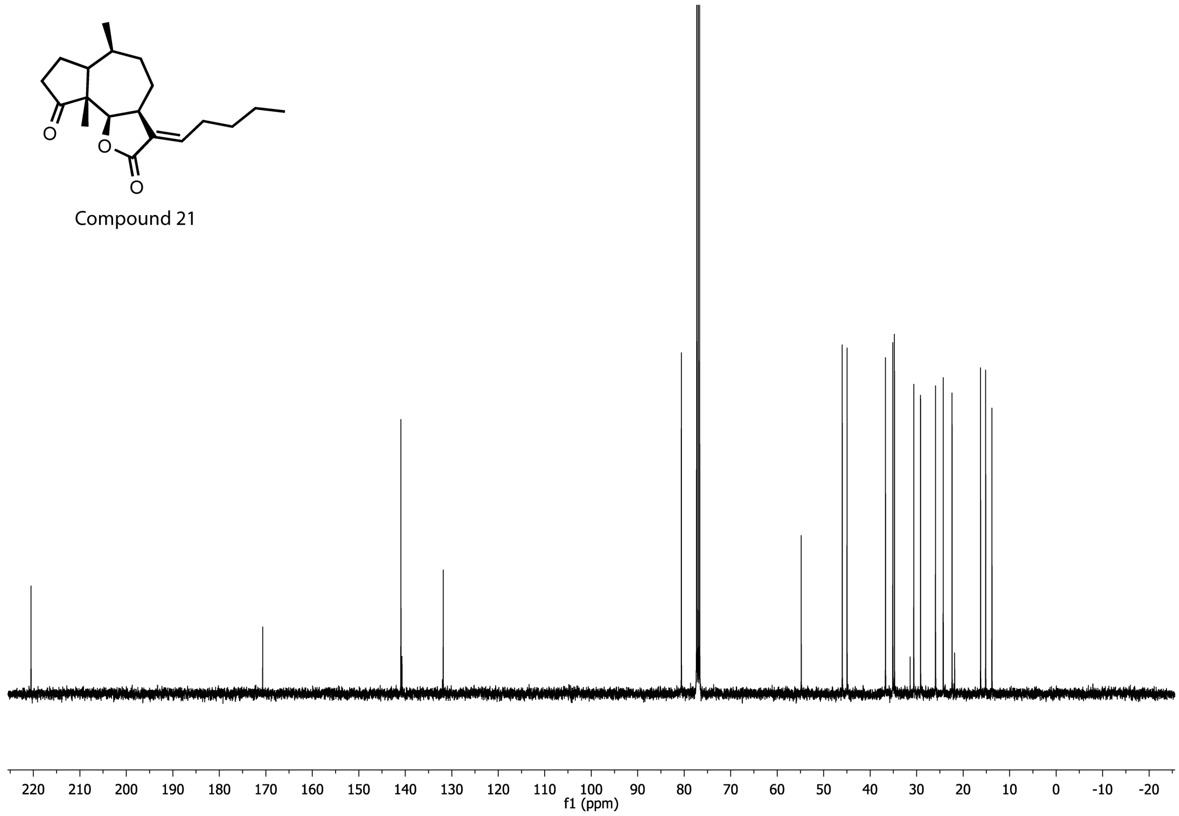

NOESY

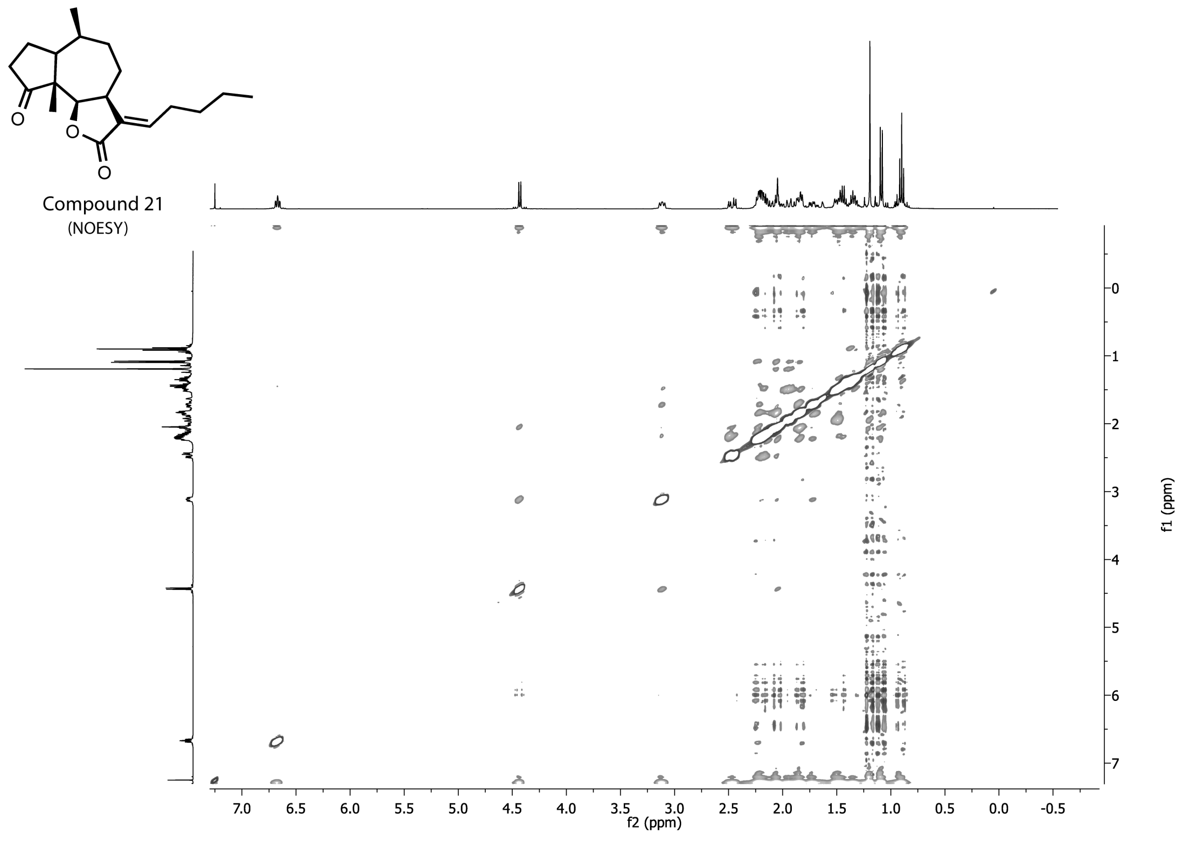

^{1}H-NMR

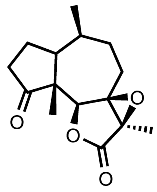

Compound 23

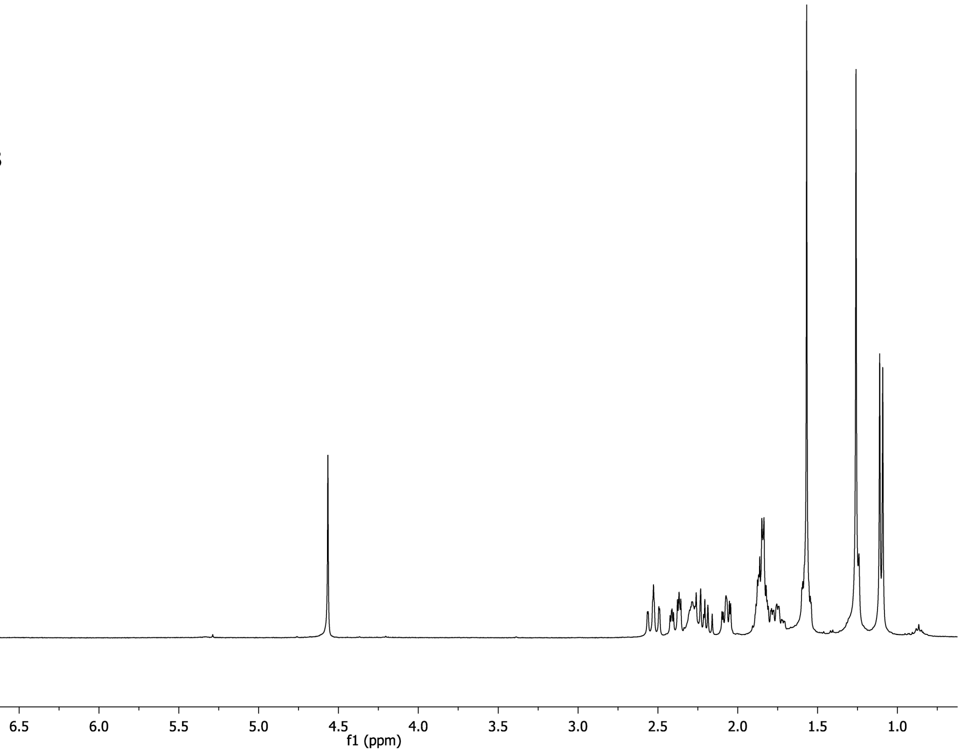

^{13}C-NMR

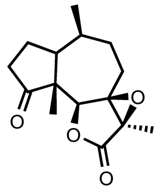

Compound 23

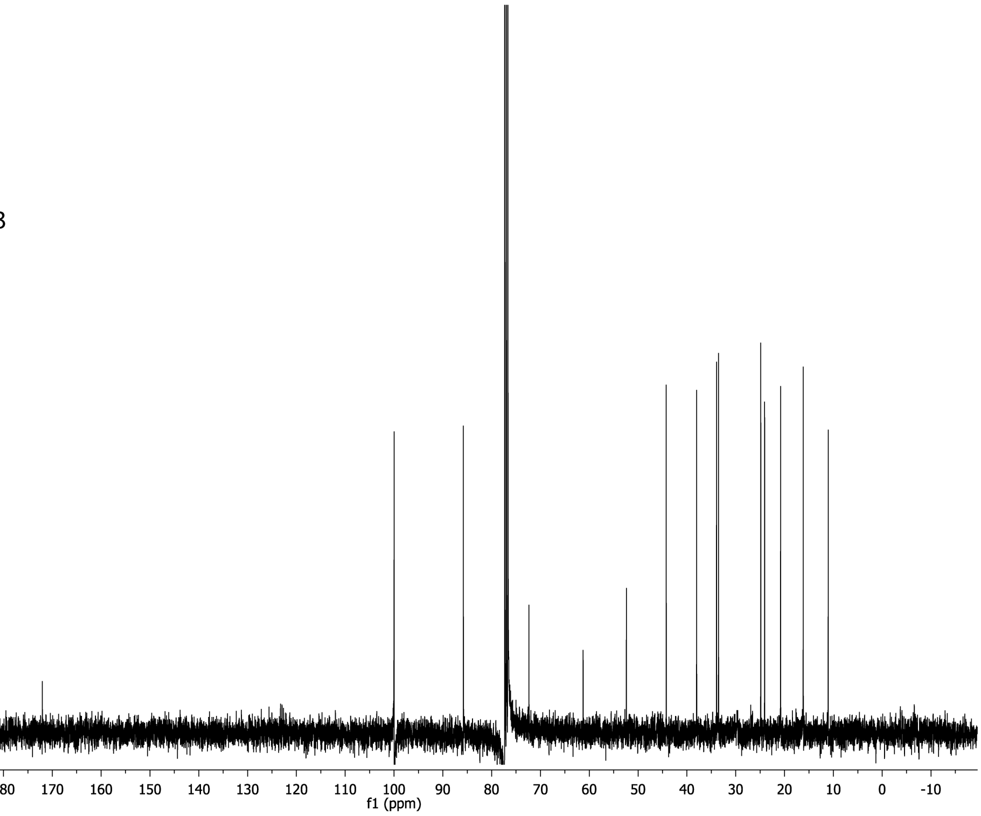

NOESY

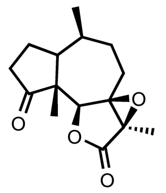

Compound 23
(NOESY)

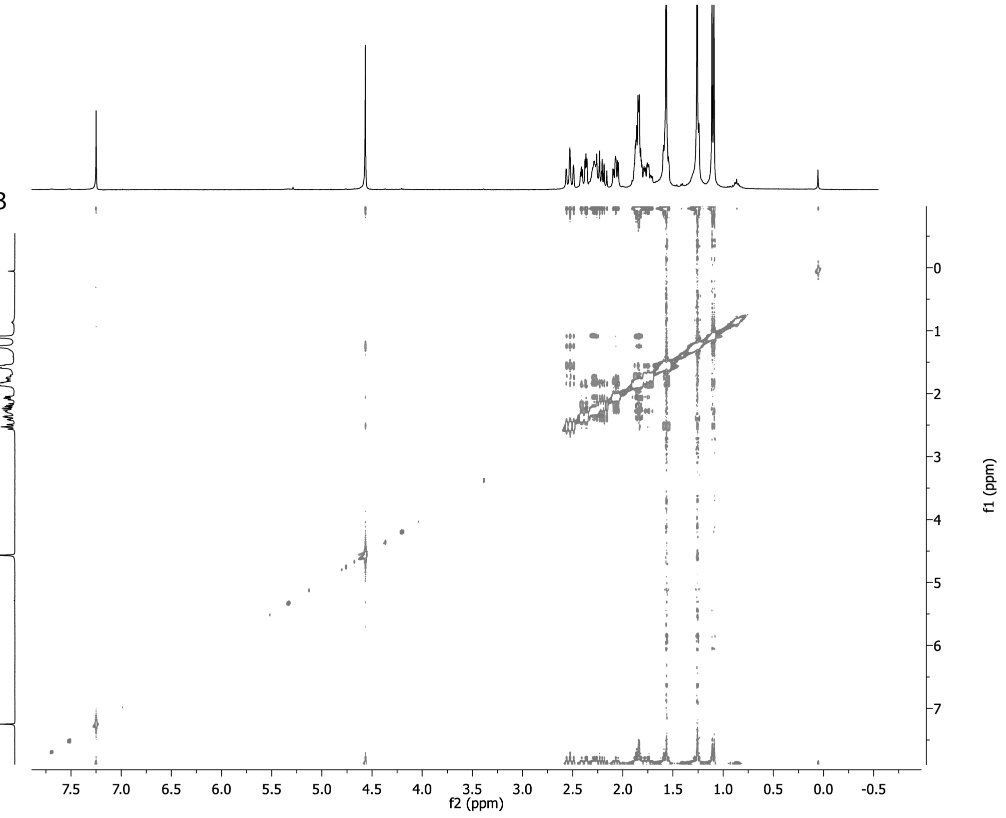

^{1}H-NMR

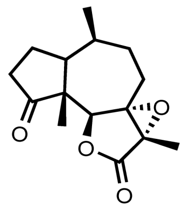

Compound 24

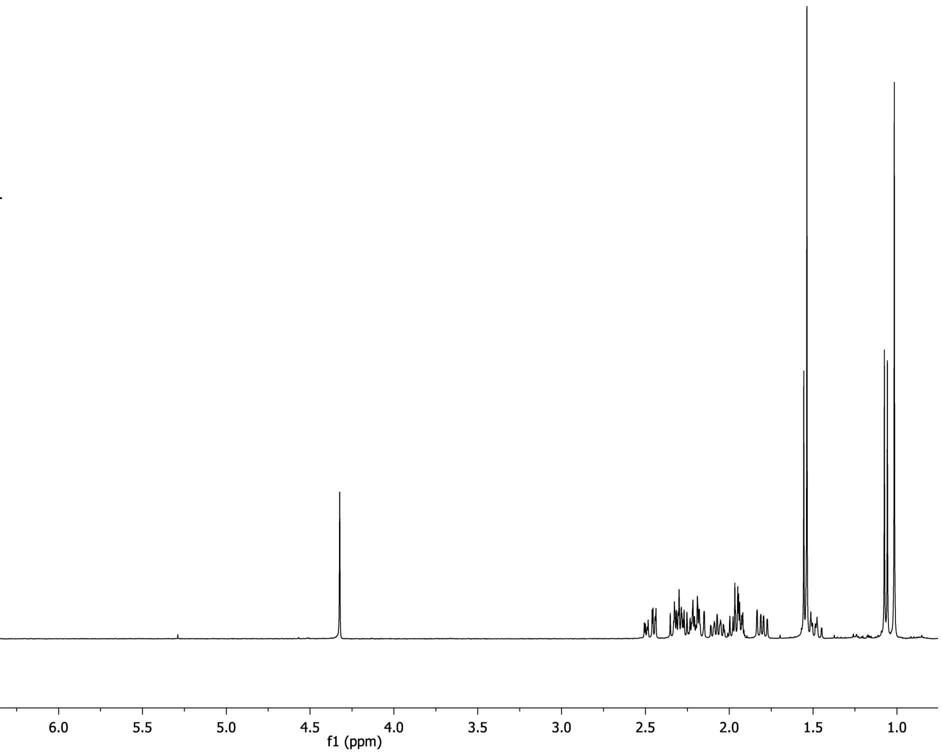

^{13}C-NMR

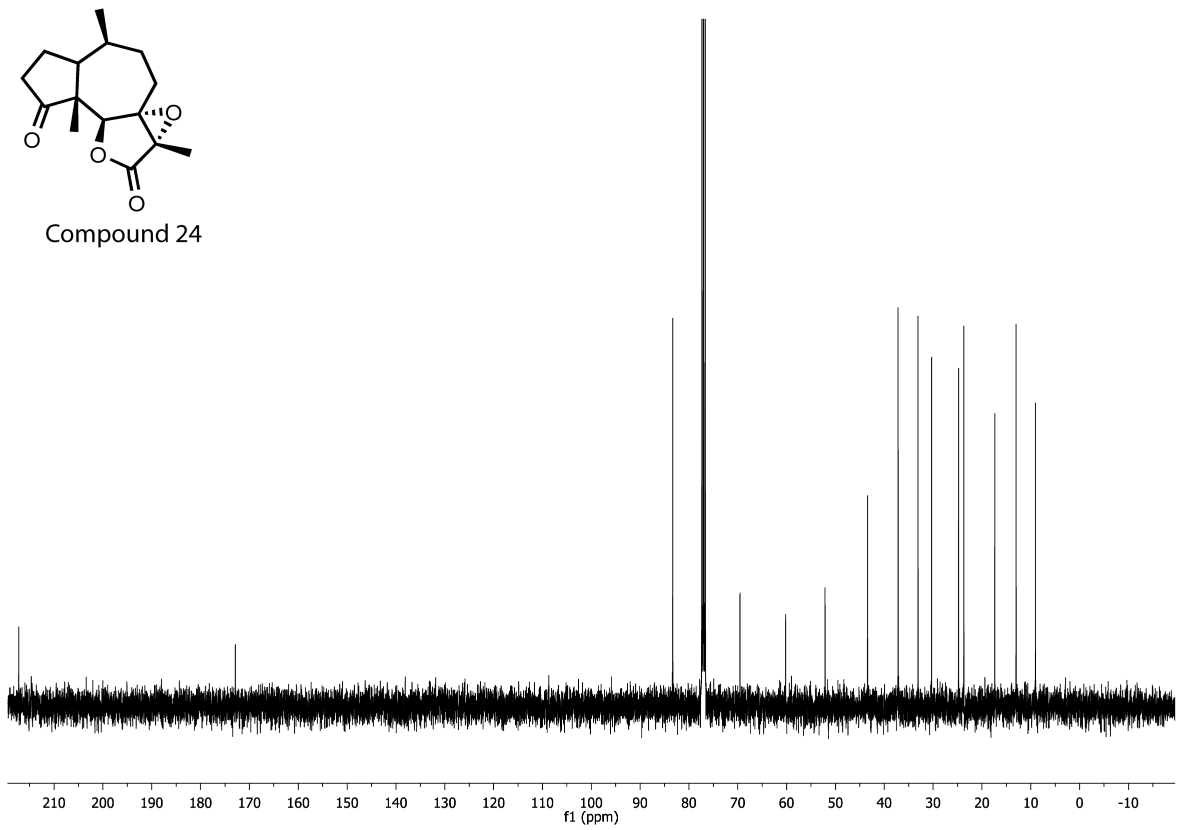

NOESY

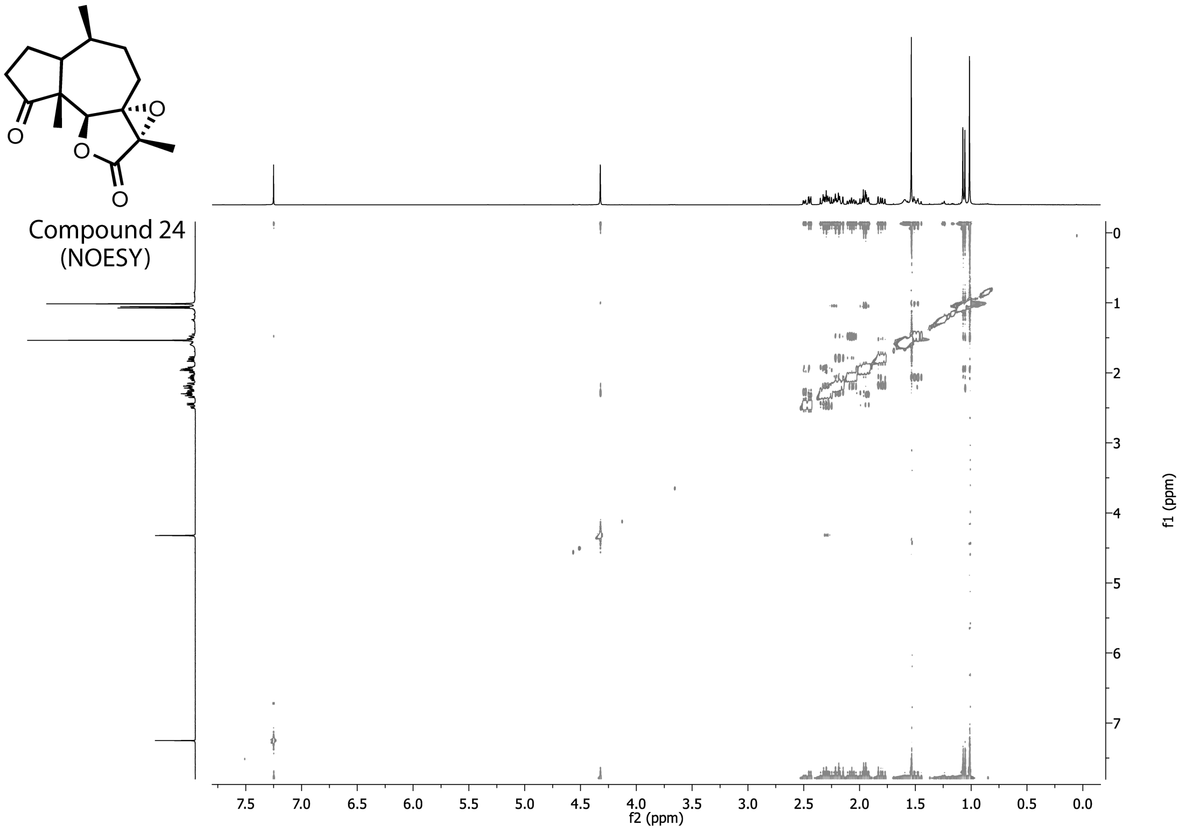

^{1}H-NMR

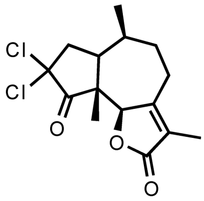

Compound 25

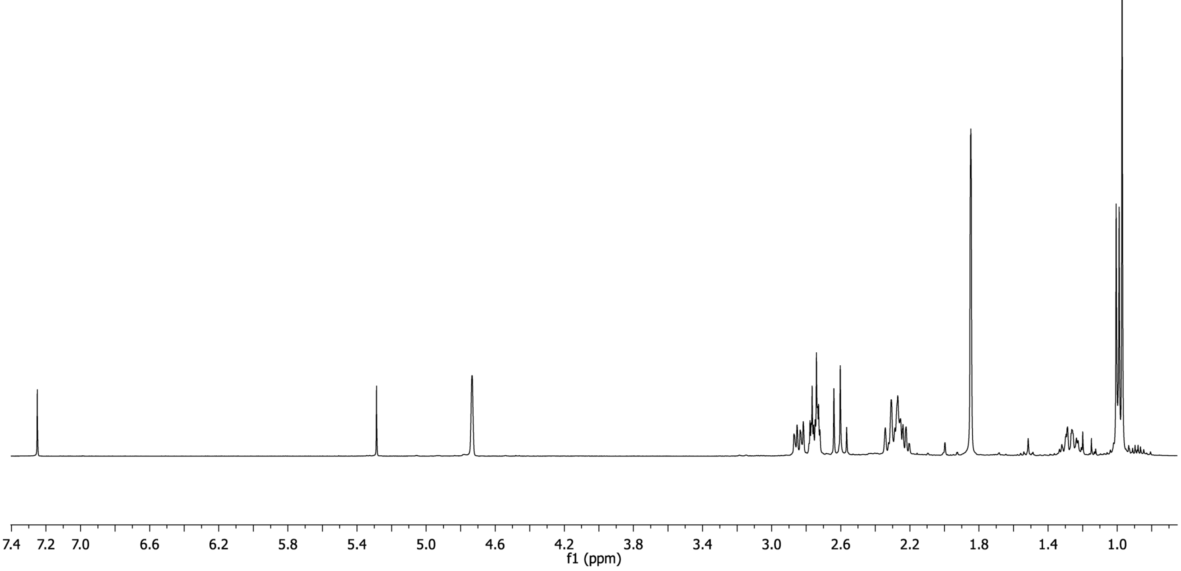

^{13}C-NMR

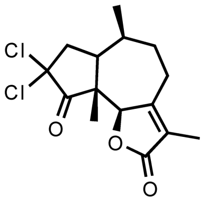

Compound 25

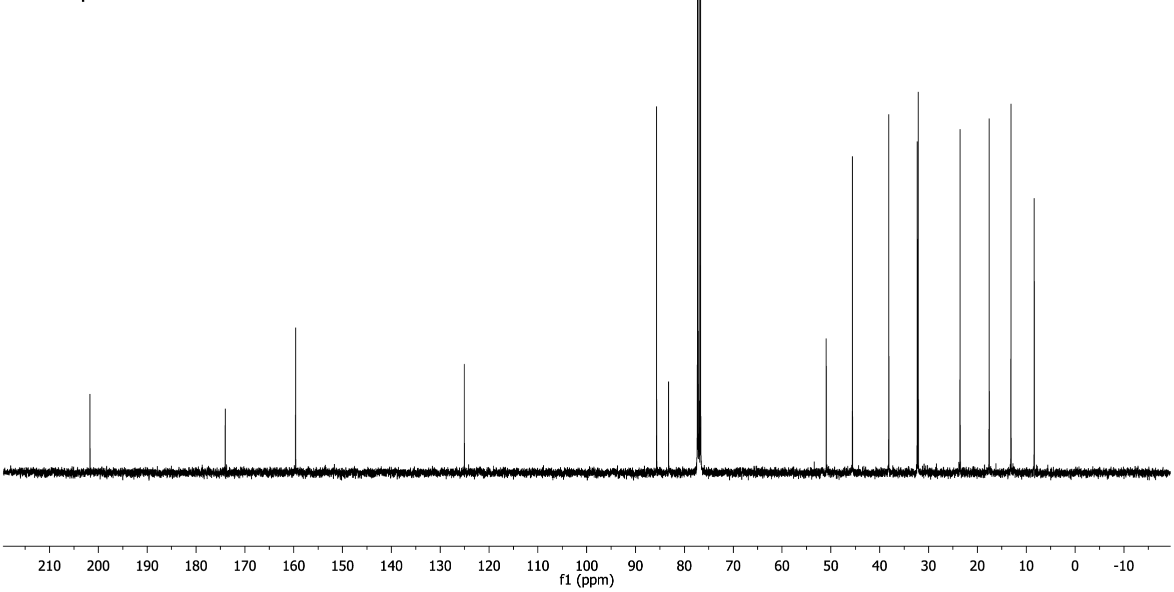

[13]H-NMR

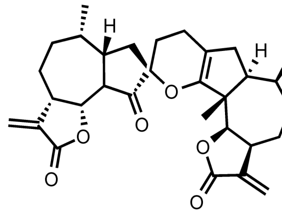

Compound 26

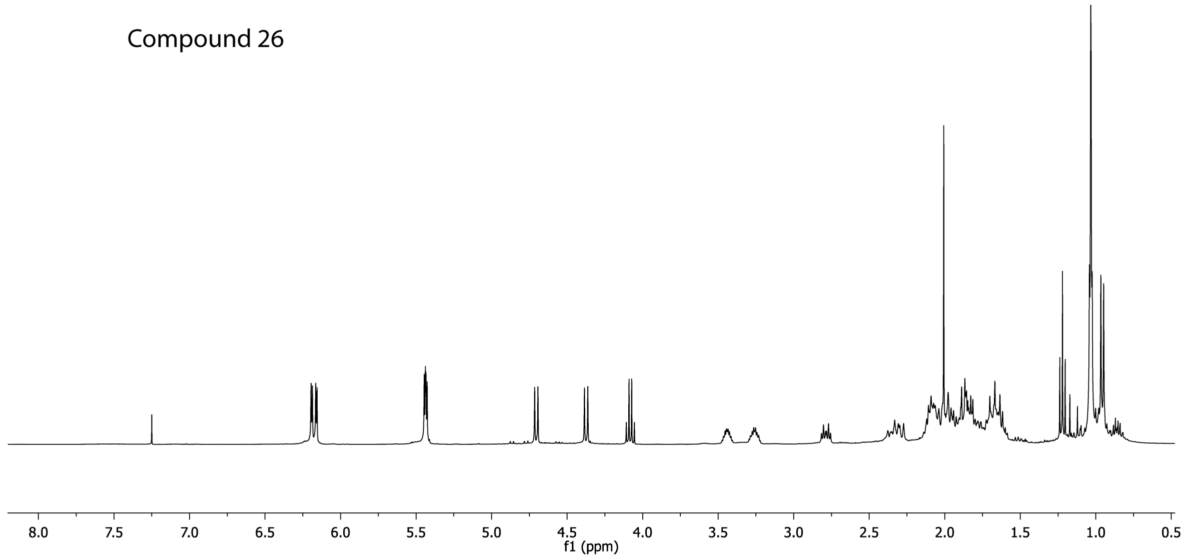

^{13}C-NMR

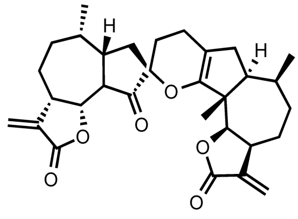

Compound 26

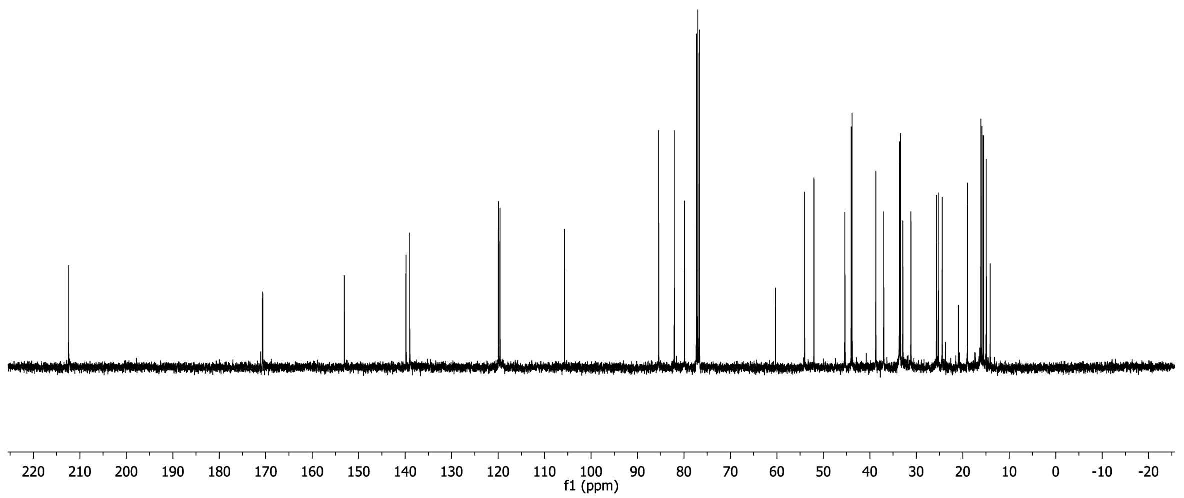

NOESY

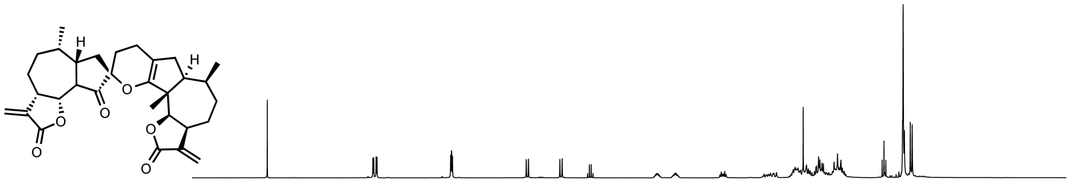

Compound 26

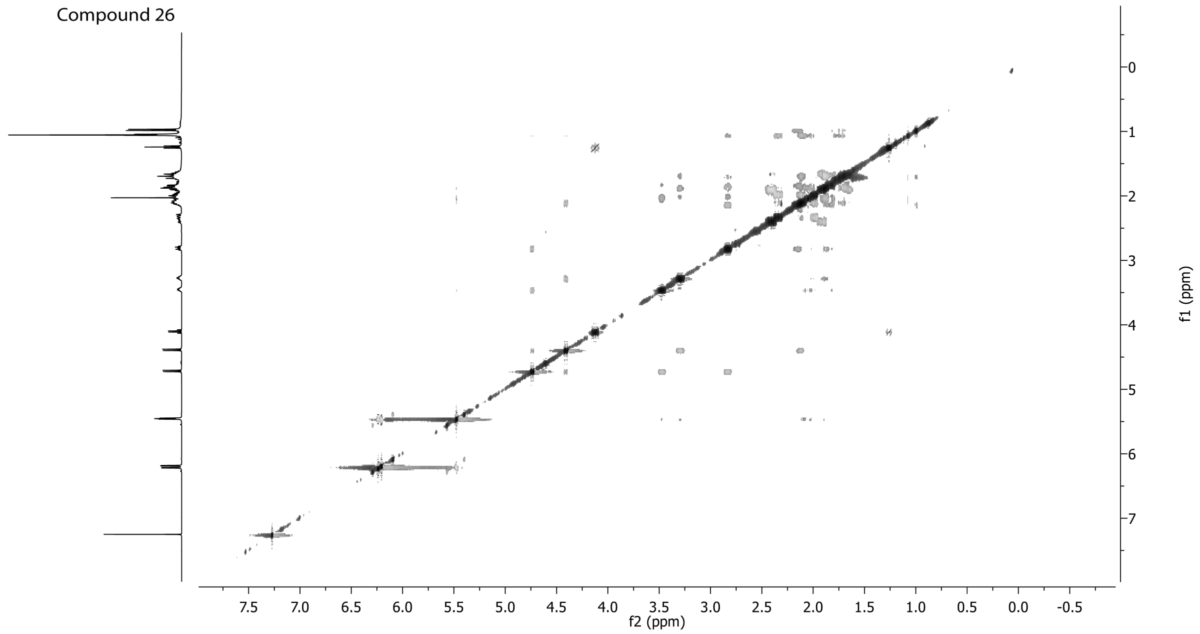

Supplement: S1 File — NMR spectra of compounds 11, 19, 21, 23, 24, 25 and 26. Compound 11. 1H-NMR 13C-NMRCompound 19. 1H-NMR 13C-NMRNOESYCompound 21. 1H-NMR 13C-NMRNOESYCompound 23. 1H-NMR 13C-NMRNOESYCompound 24. 1H-NMR 13C-NMRNOESYCompound 25. 1H-NMR 13C-NMRCompound 26. 1H-NMR 13C-NMRNOESY (PDF) [file pone.0115819.s001.pdf]
