# Supplementary material for: Effect of Natural and Semisynthetic Pseudoguianolides on the Stability of NF-κB:DNA Complex Studied by Agarose Gel Electrophoresis
Source: PLoS One. 2015 Jan 23;10(1):e0115819. doi: 10.1371/journal.pone.0115819 (PMC4304792; doi:10.1371/journal.pone.0115819)
Supplement: S2 File — Table A. Dilutions prepared to test the effect of the concentration of the reaction components. Table B. Dilutions prepared to test the effect of DMSO concentration. (PDF) [file pone.0115819.s002.pdf]

Effect of Natural and Semisynthetic Pseudoguianolides on the Stability of NF-κB:DNA Complex Studied by Agarose Gel Electrophoresis

Rodrigo Villagomez, Rajni Hatti-Kaul, Olov Sterner, Giovanna Almanza, Javier A. Linares-Pastén

Supporting Information

S2 Tables

Table A. Dilutions prepared to test the effect of the concentration of the reaction components

	Inhibitor		NF-κB		DNA target		
Entry	Dilution factor	Concentration (mM)	Dilution factor	Concentration (ng/µL)	Dilution factor	Concentration (ng/µL)	Molar ratio NF-κB:DNA
I	1	9	1	528	1	8.5	595:1
II	0.75	6.7	0.75	396	0.5	4.2	893:1
III	0.5	4.5	0.5	264	0.25	2.1	1190:1
IV	0.25	2.2	0.25	132	0.12	1.1	1190:1

Table B. Dilutions prepared to test the effect of DMSO concentration

Entry	DMSO	Inhibitor Concentration (mM)	NF-κB Concentration (ng/µL)	DNA target Concentration (ng/µL)	Molar ratio NF-κB:DNA
1	10%	9	528	8.5	595:1
2	20%	9	462	8.5	521:1
3	30%	9	396	8.5	446:1
